# Mapping the characteristics of training for vocal conditioning of singers: a scoping review

**DOI:** 10.1590/2317-1782/e20250373en

**Published:** 2026-07-03

**Authors:** Pamela Carvalho, Priscila Oliveira, Denis Batista, Flávia Viegas

**Affiliations:** 1 Departamento de Formação Específica em Fonoaudiologia, Universidade Federal Fluminense – UFF - Nova Friburgo (RJ), Brasil.; 2 Departamento de Fonoaudiologia, Universidade Federal da Paraíba – UFPB - João Pessoa (PB), Brasil.; 3 Programa de Pós-graduação em Modelos de Decisão e Saúde, Universidade Federal da Paraíba – UFPB - João Pessoa (PB), Brasil.; 4 Dr Liang Voice Program, The University of Sydney - Sydney (NSW), Australia.

**Keywords:** Singing, Scoping Review, Voice Quality, Voice Training, Voice

## Abstract

**Purpose:**

To map and characterize intervention strategies for the vocal conditioning of singers without vocal complaints.

**Research strategies:**

Scoping review based on the question: How are the intervention strategies used for vocal conditioning of singers characterized? Searches were conducted in the following sources: LILACS, MEDLINE, Embase, Scopus, Cochrane Library, Web of Science, Google Scholar, medRxiv, ProQuest, Journal of Singing, and the Brazilian Digital Library of Theses and Dissertations.

**Selection criteria:**

Studies with vocal or respiratory training of singers without vocal complaints, of any musical genre, over 18 years of age, were included. Studies with children, other voice professionals, participants with laryngeal and vocal alterations, voice rehabilitation, exclusively indirect approach or those that did not focus on vocal conditioning were excluded.

**Data analysis:**

The results were summarized in tables and charts.

**Results:**

A total of 12,358 studies were identified, eight of which met the eligibility criteria. The approaches focused on respiratory muscle strength and Semi-occluded Vocal Tract Exercises stood out. The number of repetitions together with monitoring of the total execution time was the most used. The duration of the interventions ranged from three weeks to six months and most of the training programs were conducted by speech-language pathologists and professionals from other areas. There was a predominance of self-assessments, with mostly positive results.

**Conclusion:**

Variability was found in the intervention strategies used. The focus on simultaneous work between breathing and voice stood out, with respiratory muscle strength and Semi-occluded Vocal Tract exercises being the most used techniques.

## INTRODUCTION

Singing properly involves several factors. However, doing it for long periods and repeatedly requires a lot of preparation and discipline from the artist^(1)^. Singers are differentiated occupational voice users. Their professional performance requires high demand, specificity, and excellent voice quality. Such characteristics place them in a category considered as "vocal elite"^(2)^. High vocal demands expose singers to greater susceptibility to vocal disorders, highlighting the need for constant attention and care with this tool of their trade. This care must go beyond guidelines alone, since specific conditioning is necessary for them to withstand prolonged voice use^(3)^. Training focused on muscle efficiency and fatigue resistance is essential for artists who perform for several hours^(4)^.

The number of performances by singing voice professionals has increased, as well as the demands regarding their performance, which are becoming increasingly diverse^(5)^; thus, they have been compared to athletes^(1,4,6,7)^. Whether in sports or vocal art, a higher level of performance is expected. The concept of high performance refers to success beyond what is expected, with consistent results for prolonged periods, not being an inherent characteristic of the individual, but the consequence of a specific set of practices. In addition to maintaining the stability of high performance for long periods, those who achieve high performance also need to master many areas that are somehow related to their careers^(8)^. Vocal conditioning is one of the skills that high-performance singers need, through which they may be able to support the growing demand for performances in concerts, rehearsals, and recordings, presenting satisfactory and consistent long-term results.

The voice, like all body movements, is the product of various neuromuscular actions^(9,10)^. In this sense, and especially when exposed to excessive use without proper technique, it can suffer negative effects, such as lack of resistance, fatigue, and effortful production^(11,12)^. Vocal conditioning is the preparation of the muscles involved in voice production to optimize performance, provide resistance to fatigue, and favor muscle recovery. In other words, its purpose is to provide conditions for these muscles to adapt to the needs and requirements imposed on spoken and sung voice professionals, thus reducing the risks of injuries or adversities in their performance. This implies a regular exercise program. It is recommended that a conditioning program be applied to vocally healthy individuals^(4)^. However, there are gaps in scientific production regarding the use of effective intervention strategies to increase these artists’ vocal performance.

Scoping reviews have proven to be useful for recognizing evidence and providing a broad overview of a topic^(13)^. This type of literature review aims to map the fundamental concepts of an area of knowledge with diversified study designs, with reliability and quality^(14-17)^. The objective of this review is to identify and systematize knowledge of vocal habilitation to improve the performance of professionals who work with the vocal conditioning of singers. Thus, the scoping review is the most appropriate method for this research. This review aimed to map and characterize the intervention strategies available in scientific literature for the vocal conditioning of singers without vocal complaints.

## METHOD

### Protocol design and registration

This scoping review was conducted using the Joanna Briggs Institute (JBI) methodology for scoping reviews^(18)^ and described according to the Preferred Reporting Items for Systematic Reviews and Meta-Analyses – Extension for Scoping Reviews (PRISMA-ScR)^(19)^. The review protocol was registered in the Open Science Framework^(20)^.

Initially, a preliminary search was conducted in electronic databases and in platforms that record review protocols in progress. No systematic or scoping reviews with the same objective were identified.

The present scoping review was guided by the following research question: "What are the characteristics of intervention strategies for vocal conditioning of singers?". The PCC structure (population, concept, and context) was used to outline the study, namely: the population was singers without vocal complaints, of any musical genre, and over 18 years old; the concept was the characteristics of the interventions; and the context was vocal or respiratory training focused on conditioning.

### Eligibility criteria

The review considered experimental and quasi-experimental studies, including randomized controlled trials, non-randomized controlled trials, before and after studies, and case series. It excluded studies with children, occupational voice users other than singers, participants with laryngeal and voice alterations, studies describing interventions aimed at voice rehabilitation, with an exclusive approach to indirect intervention, or that did not focus on vocal conditioning. Observational studies, reviews, letters to the editor, editorials, books, book chapters, and abstracts published in the annals of scientific events were also not considered.

### Data search and selection

An initial search was conducted in the PubMed database via MEDLINE to identify articles on the topic. The words contained in the titles and abstracts of the relevant articles, as well as the indexing terms used to describe the articles, were used to develop an initial search strategy, based on the PCC elements. This search strategy was improved from the first articles found, considering all keywords and indexing terms identified. Indexing vocabularies from PubMed (Medical Subject Headings – MeSH), Embase (Emtree), and the Health Sciences Descriptor Library (DeCS) were used, as well as free terms based on the PCC model. The strategy was adapted for each database or information source, as presented in Appendix A, covering LILACS (VHL), MEDLINE (PubMed), Embase, Scopus, Cochrane Library, and Web of Science. A manual search was also performed in the reference lists of the articles selected from the electronic databases, as well as an additional search in Google Scholar (100 first studies), MedRxiv, ProQuest, Brazilian Digital Library of Theses and Dissertations (BDTD), and Journal of Singing. The latter was included due to its high specific impact on the target population of the study. No language or period restrictions were applied to increase the sensitivity of this research. The searches were carried out on May 26, 2025.

**Figure 1:**
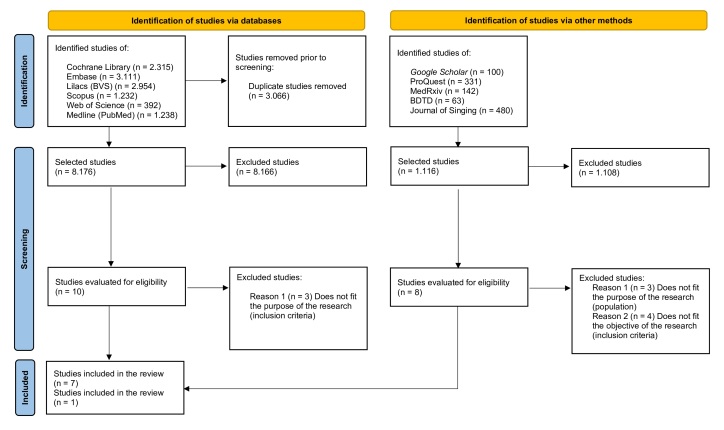
PRISMA Workflow Chart^(22)^

The selection of relevant studies involved two stages: screening of titles and abstracts and full reading of the studies. Before starting the selection, the two reviewers were calibrated based on the first 25 articles retrieved from the PubMed database (via MEDLINE); their agreement index, calculated by the Cohen's Kappa Test, was 100% (k = 1). After calibration, all citations identified in the sources of evidence were pooled and uploaded to Rayyan^(21)^, removing duplicates. The sources of information whose format is incompatible with the software in question were searched manually. The reviewers screened the titles and abstracts, observing the inclusion criteria for the review. The full texts of the selected citations were evaluated in detail in relation to the exclusion criteria. This entire process was carried out independently and blindly. The disagreements between the reviewers were resolved by consensus. After reading articles in full, a manual selection was made from the reference lists of those included in the review. The search results and study inclusion process are presented in a flowchart (Figure 1).

Two reviewers extracted data independently and blindly, using a data extraction spreadsheet previously developed for this study. After extraction, the data were compared, and disagreements were resolved by consensus.

As described in Appendix B, the following information was collected: year, authorship, country of publication, title, journal, objective, study design, sample characteristics (sample size, sex, and age), exercise/technique used, dosage, frequency (performance of exercises and sessions), total duration of the intervention, professional who applied it, outcome measures, results, conclusion, and limitations/implications for the future.

### Data analysis and synthesis

The results of this review were presented by means of relative (%) and absolute (n) frequencies, tables, and charts in a descriptive synthesis of the findings.

## RESULTS

A total of 12,358 studies were identified from searches in electronic and manual databases, and 3,066 duplicates were removed. Thus, 9,292 articles were screened by reading titles and abstracts; 9,274 of them were excluded because they did not meet the inclusion criteria, totaling 18 studies eligible for reading in full. Ten were excluded for not presenting vocal interventions or addressing participants’ complaints or research issues other than the subject of this review. The remaining eight studies were considered eligible for this review. The PRISMA flowchart (Figure 1) shows the inclusion and exclusion of the selected articles.

The studies selected for analysis were published between 1995 and 2024, with most of them recorded in the last 4 years, with three studies (37.5%). Three of the eight studies were conducted in the United States of America (37.5%), two in Brazil (25%), one in Germany (12.5%), one in South Korea (12.5%), and one in Turkey (12.5%). The journals with the most publications were the Journal of Voice and *Distúrbios da Comunicação*, with two publications each (25% each); Sprache · Stimme · Gehör, Journal of Singing, Journal of Religion and Health, and Yonsei Medical Journal accounted for one publication each (12.5% each). Various study designs were identified – before and after studies were the most prevalent type (75%; n = 6), followed by quasi-experimental (12.5%; n = 1) and experimental (12.5%; n = 1) studies (Chart 1).

**Chart 1:** Characteristics of the included studies

**Author**	**Title**	**Objective of the Study**
Sezin et al.^(23)^	Effectiveness of a Voice Training Program for Religious Officials in Turkey	Examine the effectiveness of a voice training program designed for Islamic religious authorities
Ferreira et al.^(24)^	Vocal and Respiratory Conditioning Program (CVR II): new proposal for voice professionals	Describe a vocal and respiratory conditioning program
Ferreira et al.^(25)^	Vocal and Respiratory Conditioning Program (CVR): intervention proposal for voice professionals	Describe an intervention proposal that includes vocal and respiratory muscle training
Sandage and Hoch^(26)^	Training Considerations for Recital Performance: Framing Vocal Dose in a Fatigue Resistance Training Model	Understand the workload required for a given vocal effort. Understand how the vocal dose, which quantifies vocal effort, can be used to guide the training and preparation of singers, aiming to reduce perceived fatigue during and after performances
Ray et al.^(27)^	Effects of Respiratory Muscle Strength Training in Classically Trained Singer	To understand whether it is possible to increase respiratory muscle strength with progressive threshold training and whether the increase in respiratory muscle strength had a measurable effect on voice outcomes
Bengtson-Opitz^(28)^	Vocal Anti-Aging A Fitness-Programme for the Aging Voice	To present a vocal prevention program that seeks to improve vocal aptitude by delaying voice aging in the elderly
Nam et al.^(29)^	Specially programmed respiratory muscle training for singers by using respiratory muscle training device (Ultrabreathe^®^)	To examine pulmonary function, maximal inspiratory pressure (MIP), maximal expiratory pressure (MEP), and maximal phonation time (MPT) after specially programmed respiratory muscle training
Sabol et al.^(30)^	The Value of Vocal Function Exercises in the Practice Regimen of Singers	To investigate the effects of isometric-isotonic vocal function exercises on vocal production parameters

The sample size ranged from one to 34 participants. Considering the studies that provided demographic data, the male sex was predominant, with a total of 43 men and 25 women, and the participants’ ages ranged from 20 to 80 years, with a mean of 23 to 43.7 years (Table 1).

**Table 1:** Characteristics of the strategies

**Author**	**Sample**	**Exercise/Technique**	**Dosage**	**Frequency of Performing the Exercises**	**Frequency of Sessions**	**Total Duration of Intervention**	**Professional who applied**
Sezin et al.^(23)^	N = 34	Posture, breathing, vocal warm-ups, resonance and projection	INA	INA	32 sessions over 8 weeks (8 sessions with indirect voice therapy approach and 24 sessions with direct voice therapy approach)	8 weeks	INA
	Sex = M						
	Age = CG - average 44.3						
	EG - average 43.7						
Ferreira et al.^(24)^	N = 3 (1 singer)	1. Respiron Athletic 2^®^	1. 2 sets of 10 and 12 repetitions	Daily 1 time a day	1 meeting per week lasting 1h30	11 weeks	2 speech therapists conducted the proposal and students from the undergraduate courses in speech therapy and physiotherapy, as well as their advisors, professors from each course, also followed the process
		2. Sounded blowing exercise on the LaxVox^®^ alternating between modulated, monotone and hyperacute					
			2. 10 repetitions with 15 seconds				
	Sex = M						
			3. 10 repetitions with 6 and 9 seconds				
	Age = average 29.6						
		3. Sounded blowing exercise on the New Shaker^®^ alternating between modulated, monotone, habitual and hyperacute					
Ferreira et al.^(25)^	N = 3 (1 singer)	1. Respiron Classic^®^	1. 2 sets of 10, 12 and 15 repetitions	Daily 2 times a day	1 meeting per week	8 weeks	The program was conducted by a speech therapist accompanied by 2 undergraduate students in speech therapy and physiotherapy, and the students were guided by teachers from their respective courses
		2. Sounded blowing exercise on the milkshake straw alternating between hyperacute, monotone and modulated					
			2. 10 repetitions in 12 seconds				
	Sex = M						
			3. 10 repetitions at 12 and 14 seconds				
	Age = INA						
			4. 10 repetitions at 14 and 16 seconds				
		3. Sounded blowing exercise on the LaxVox^®^ tube alternating between hyperacute, monotone and modulated					
			5. 10 repetitions in 12 seconds				
		4. Sounded blowing exercise on the lollipop straw alternating between hyperacute, monotone and modulated					
		5. Sounded blowing exercise on the lollipop straw alternating between hyperacute, monotone and modulated					
		* Vocal exercises in approximate time between two and three minutes					
Sandage and Hoch^(26)^	N = 1	1. Musical preparation with sheet music	3. 60 minutes per day (20-30 minute sessions)	1. Mental Practice + Sheet Music Study - 7 days a week	INA	1. 6 months	Singing teacher and speech therapist. Singer and singing teacher
						2. and 3. 4 months	
						4. and 5. 1 month	
		2. Technical work: humming, lip trills, straw phonation, neutral syllables, and singing on vowels					
	Sex = M						
				3. 5 days a week			
				4. Essay with the accompanist + regular vocal practice - 2 days a week			
			5. 2 to 3 plays				
	Age = INA	3. Physical practice					
		4. Essay with the accompanist + regular vocal practice					
		5. Performing the entire performance in formal concert attire					
Ray et al.^(27)^	N = 6	1. IMT - adjusted to 80% of MIP	5 sets of 5 repetitions (1-3 minute intervals between each set)	Daily 1 time a day	1 meeting every 5 to 10 days	3 to 7 weeks	INA
		2. EMT - adjusted to 80% of MEP					
	Sex = F						
	Age = average 28						
Bengtson-Opitz^(28)^	N = 10	1. Body/posture/breathing – awakening the sensitivity and flexibility of the rectus abdominis muscle and strengthening of the intercostals	10 minutes	Daily 3 times a day	6 meetings over 6 weeks lasting 90 minutes	6 meetings	Future singing teachers supervised by the author of the course/method and a sports therapist takes over the work with the body and posture
	Sex = INA						
	Age = between 70 and 80 years old						
		2. Embouchure/articulation of vowels and consonants (sound modeling):					
		b. Lips: rounded and mimic vowels					
		c. Language: "connecting rod exercise" silently and with the vowels /i/, /a/					
		d. Soft palate: yawns, loud laughter, and closing sounds /k/, /g/, /ng/, /ʃ/					
		3. Consonants – plosive (voiced and unvoiced) and fluent (long or short)					
Nam et al.^(29)^	N = 5	Various exercises with the Ultrabreathe^®^	1. 5 to 10 minutes	1 and 3. Daily 7 to 10 times a day	INA	Approximately 2 months	INA
			2. 60 to 90 minutes				
			3. approx. 10 minutes				
	Sex = F						
	Age = average 23						
Sabol et al.^(30)^	N = 20	1. Vowel /i/ sustained in MFT	2 repetitions (15-20 minutes)	Daily 2 times a day	3 times a week	4 weeks	Principal investigator of the study and graduate students in speech-language pathology who have been trained in proper technique by the third plaintiff who is a certified and licensed speech pathologist with a specialization in voice
		2. Ascending glissando with the vowel /o/.					
	Sex = 6M and 14F						
		3. Descending glissando with the vowel /o/					
	Age = EG - average 30						
		4. Sustained musical notes in MFT with the vowel /o/ (2 times)					
	CG - average 28						

**Caption:** M = male; F = female; CG = control group; EG = experimental group; INA = information not available; MFT = maximum phonation time; IMT = inspiratory muscular training; EMT = expiratory muscle training; MIP = maximum inspiratory pressure; MEP = maximum expiratory pressure

The approaches that aimed at respiratory muscle strength stood out among the exercises/techniques used. This type of exercise was observed in five studies^(24,25,27-29)^, followed by semi-occluded vocal tract exercises (SOVTE), with four studies^(24-26,28)^. Four studies included indirect approach strategies with vocal care guidelines^(23,25,26,30)^, three studies^(23,26,28)^ used resonance exercises and humming, and two studies^(26,30)^ used vowels sustained in a comfortable tone, glissando, or musical melody. Various devices were identified, including elastic bands, balls, straws, dumbbells, mirrors, corks, respiratory muscle strength training devices, tubes, incentive spirometers, and positive expiratory pressure (PEP) devices (Table 1).

The dosage varied; the most commonly used was the number of sets and repetitions, observed in four studies (50%). Three studies (37.5%) did not provide details on this variable. The frequency of sessions ranged from three per week to longer periods, 10 days apart. The total duration of the intervention ranged from 3 weeks to 6 months. Regarding the professionals who applied the training, the most cited were undergraduate or graduate speech-language and audiology students, supervised by their respective professors/advisors, present in three studies (37.5%). In some studies, they worked in partnership with students from other areas, such as physical therapy (Table 1).

Regarding the outcome measures, just over half of the studies (n = 5; 62.5%) used vocal self-evaluation. Aerodynamic measurements (n = 4; 50%), acoustic measurements (n = 4; 50%), and respiratory muscle strength measurements (n = 3; 37.5%) were also mentioned. The least used methods were vocal frequency range analysis (n = 2; 25%), auditory-perceptual evaluation (n = 1; 12.5%), intensity measurements (n = 1; 12.5%), vocal dose (n = 1; 12.5%), pulmonary function (n = 1; 12.5%), and videostroboscopy (n = 1; 12.5%) (Chart 2).

**Chart 2:** Outcome measures, results and conclusion

**Author**	**Outcome Measures**	**Results**	**Conclusion**
Sezin et al.^(23)^	1. Acoustic measurements: f_o_, jitter, shimmer and HNR	1. There was a significant reduction in shimmer (from 1.76% to 1.41%) and jitter (from 0.27% to 0.21%). The HNR increased significantly (from 25.44 to 26.76). There was no significant variation in f_o_	Beneficial results were observed from a voice education and training program designed for Islamic religious officials
	2. Self-evaluation: VHI-C, VFI and V-RQOL		
		2. VHI-C and VFI - a significant decrease in the perception of vocal handicap (from 18.00 to 12.17) and in the symptoms of vocal fatigue reported (from 29.05 to 19.82) was reported. V-RQOL - Voice-related quality of life improved significantly (from 68.00 to 86.76). The CG did not present significant changes in any of the parameters evaluated, indicating that the improvements observed in the EG were attributed to the vocal training program	
Ferreira et al.^(24)^	1. Auditory-perceptual analysis: vowel /a/ emitted 3 times and spontaneous speech - pitch, loudness, resonance pneumophonoarticulatory coordination, speech rate, articulation and modulation	1. Increased, decreased or stabilized	The use of respiratory encouragers as a proposal to improve vocal and respiratory conditioning and the partnership with physiotherapy are presented and recommended for a better understanding and consequent care of voice and breathing issues in voice professionals
		2. Increased	
		3. Greater vocal projection, better possibility of producing higher pitched sounds and more vocal flexibility. VFI - decreased fatigue and vocal restriction for 2 participants and vocal discomfort for 1 of them, VHI-10- even before there was no disadvantage. Even lower registration for 2 participants after training and EASE-BR subscale scores varied among participants	
	2. Aerodynamic measurements: MFT		
	3. Self-evaluation: directed questions, VAS, VFI, VHI-10 and EASE-BR		
	4. Respiratory measures: respiratory muscle strength (MIP and MEP) and RME	4. Considerable improvement in respiratory indices and decrease in the perception of respiratory effort	
Ferreira et al.^(25)^	1. Self-evaluation: directed questions, system for recording scores from 0 to 10 regarding voice quality and phonatory comfort, VHI and EASE-BR	1. Improvements in self-perception, motivation, vocal and respiratory control, and phonatory comfort have been reported. There was a decrease in the feeling of fatigue. It was observed that the improvement in breathing did not have immediate repercussions on the voice	The proposal proved to be viable and promising, especially when performed continuously and with gradual progression of exercises. The integration of the areas of speech therapy and physiotherapy is beneficial to optimize vocal and respiratory conditioning. The program presented has the potential to record positive effects
Sandage and Hoch^(26)^	1. Self-evaluation: VFI and PPE	1. VFI - measurements were higher after each singing attempt compared to the pre-singing measurement; however, they decreased between trials as hypothesized. PPE - did not change by more than a few points across the four trials	The lower vocal fatigue perceived throughout rehearsals for the recital provides preliminary evidence of a conditioning training effect to meet the performance demands of this specific repertoire and recital in general
	2. Vocal dose measurements.		
		2. Vocal dose - was ambiguous for the two measured moments, establishing a consistent vocal dose in the last two rehearsals (2954.56 meters during rehearsals and 3098.18 meters during the recital, with an average sound intensity of 93 dB and 94 dB)	
Ray et al.^(27)^	1. Respiratory measures: MIP and MEP	1. There was a significant increase in MIP and MEP measurements. (IMST (phase B) MIP 37%, 101% and 118% / MEP 23%, 45% and 130% - EMST (phase C) MEP 70%, 28% and 137% / MIP 16%, 33% and 34% - IMST and EMST (phase D) MIP 63% / MEP 104%)	The results showed that the singers increased respiratory muscle strength, both in the form of inspiratory (IMST) and expiratory (EMST) training, after completing the training program. There were no consistent changes in aerodynamics and voice measures among the subjects, although some individual changes were observed
	2. Acoustic measurements: Phonetogram: f_o_ range, intensity range (SPL) and airflow for each tone		
		2. F_o_ range - 3 participants showed increased pitch range. None of the participants decreased the pitch amplitude during the training. Intensity Range - both increase and decrease in SPL were recorded. Airflow - Measurements were inconsistent	
Bengtson-Opitz^(28)^	INA	All participants experienced a clearly audible and noticeable improvement with improved articulation, flexibility, and pitch range	"Anti-Aging for the Voice" is not a magic solution, but rather an ongoing process that requires practice and dedication. Maintaining the exercises and techniques learned is critical to sustaining the long-term benefits
Nam et al.^(29)^	1. Aerodynamic measurements: MPT	1. Increased significantly (from 22.3 ± 4.3sec to 31.0 ± 6.7sec)	Specially programmed respiratory muscle training can improve respiratory muscle strength and vocal function without increasing lung function. This method can be performed easily and does not require much time
	2. Respiratory measures: pulmonary function tests (flow volume curve, plethysmography - FVC, FEV in 1 second, FEV1/FVC and FEF 25 - 75%, TLC and VC, and respiratory muscle strength test (MIP and MEP)		
		2. Pulmonary function tests - were shown to be statistically similar before and after training. Respiratory muscle strength - showed a statistically significant increase (MIP from 62.2 ± 13.5 cmH2O to 78.2 ± 3.7cmH2O / MEP from 56.4 ± 7.5cmH2O to 70.0 ± 8.1cmH2O )	
Sabol et al.^(30)^	1. Acoustic measurements: f_o_, jitter, and frequency range	1. f_o_ - There were no significant differences. Jitter – The average rate increased in comfortable tone (from 0.24 to 0.31Hz) and low (from 0.26 to 0.32Hz). Frequency range - There were no significant differences between the control and experimental groups	Vocal function exercises containing the isometric-isotonic principle have been shown to have a positive effect on the phonatory systems of healthy young singers. The results indicate that the regular practice of these exercises can lead to an increase in glottic efficiency
	2. Aerodynamic measurements: MPT, phonation volume, and flow rate		
	3. Videolaryngostroboscopy		
	4. Self-evaluation: writings about the effects of vocal function exercises on their singing performance		
		2. MPT - There was an increase in the experimental group (9s comfortable tone, 11s high tone and 9s low tone). Phonation volume - the experimental group had a slight increase (from 2,739 to 2,099ml). Flow rate - the experimental group had its flow rates decreased (from 205 to 133ml/s)	
		3. There were no significant differences between the pre-and post-test	
		4. Participants reported an increase in awareness of breath control and relaxation and improvements in glissando execution. They also reported greater awareness of laryngeal and facial tensions. The three men in the experimental group complained of tense, uncomfortable and tight sensations, especially in the first week. However, they improved their maximum phonation times over the following weeks	

**Caption:** f_o_ = fundamental frequency; Jitter = percentage of vocal frequency disturbance; Shimmer = percentage of vocal intensity disturbance; HNR = harmonic-to-noise ratio; VHI-C = Vocal Handicap Index for Singing; VFI = Vocal Fatigue Index; V-RQOL = Voice-Related Quality of Life; CG = control group; EG = experimental group; MPT = maximum phonation time; VAS = visual-analogue scale; VHI-10 = Vocal Handicap Index 10; EASE-BR = Evaluation of The Ability to Sing Easily; MIP = maximal inspiratory pressure; MEP = maximal expiratory pressure; RME = respiratory muscle endurance; VHI = Voicel Handicap Index; PPE = perceived phonatory effort; SPL = sound pressure level; IMST = inspiratory muscle strength training; EMST = expiratory muscle strength training; FVC = forced vital capacity; FEV = forced expiratory volume; FEF = forced expiratory flow; TLC = total lung capacity; VC = vital capacity

The positive effects on self-evaluation were more frequent among the results obtained with the training programs (n = 5; 62.5%). There was no difference in videolaryngostroboscopy or in intensity and frequency measures (n = 1; 12,5%). No studies reported negative results (Chart 2). Most studies pointed out limitations and/or implications for the future (n = 7; 87.5%). For example, the benefits of a more personalized exercise regimen and its effects on vocal parameters^(30)^ (n = 1; 12.5%) or the effects of long-term training and the efficacy of respiratory muscle training on acoustic parameters^(29)^ (n = 1; 12.5%) were pointed out as implications for the future. Limitations included equipment that hindered the true assessment of intended measurements^(27)^ (n = 1; 12.5%), difficulties inherent to the moment of the pandemic when singers were not acting as before^(24)^ (n = 1; 12.5%), absence of laryngeal imaging, small sample size, and absence of follow-up evaluations^(23)^ (n = 1; 12,5%).

## DISCUSSION

This review mapped and characterized intervention strategies aimed at vocal training for the conditioning of singers without vocal complaints available in scientific literature. The results can support the performance of speech-language-hearing pathologists who work with singers to ensure more effective vocal behavior for these professionals.

The review identified eight studies^(23-30)^ on the vocal conditioning of singers published between 1995 and 2024. The small number of studies found in this review shows that little has been discussed about this perspective of speech-language-hearing pathology in voice in the last few decades. Studies published in the last 4 years^(23-25)^ were more frequent, demonstrating that this type of performance may be gaining visibility in the scientific field of voice.

Most studies used a before-and-after design^(24-29),^ like another review that mapped devices used in vocal therapy and training^(31).^ Studies that employ this type of design seek to verify the effects of the intervention in a single group of participants. However, they have methodological limitations, such as the lack of a control group and participant randomization. Only one source of information^(23)^ used the experimental design, which is considered the most appropriate to determine whether an intervention was effective^(32)^.

The study samples were composed of both sexes, with the majority being male, with an average age of 23 to 43.7 years. One^(28)^ of the studies that informed demographic data regarding age reported that the participants were 70 to 80 years old, but did not provide further details in this regard. This highlights the need for scientific investigations that analyze the effects of vocal conditioning programs in the different life cycles and sexes^(33)^.

The intervention groups ranged from one to 34 participants. In scientific investigations, small sample sizes can hinder adequate levels of significance and statistical power, making it impossible to generalize the results to a larger population. Sample calculations can resolve this methodological issue^(34).^

Research objectives also varied. They proposed to investigate the effects of exercises^(29,30)^; to understand whether it is possible to increase respiratory muscle strength and to verify if, in doing so, it would affect the voice^(27)^; to understand how the vocal dose can be used to guide training(25); to examine the efficacy of a training program^(23)^; and, finally, three studies presented training programs specifically focused on voice conditioning^(24,25,28)^. Research sources are scarce in the literature portraying vocal conditioning programs, which could be due to some reasons, such as the recent interest in vocal training performance among healthy singers and the limited knowledge about bioenergetic aspects, laryngeal muscle response to training, and vocal production mechanisms.

Half of the studies used strategies with a joint approach between respiratory and vocal functions^(23-25,28)^. Some studies addressed voice training^(26,30)^ or breathing^(27,29)^ alone. Similar to what was found in half of the studies present in this review, a study^(35)^ presented a conditioning program with an equal proportion of breathing and voice exercises; however, the effects of this program have not yet been investigated. At this point, it is difficult to establish clear vocal conditioning objectives in the literature, as there are still many doubts and conceptual uncertainties in this context.

Respiratory conditioning exercises were predominant, being reported in five of the eight studies^(24,25,27-29)^. This result contrasts with a study^(36)^ in which the most used approach to train individuals with healthy voices was SOVTE, focused on the voice, mainly with phonation in tubes and straws. This finding demonstrates a trend that may emerge from a greater understanding of the particularities of singer training.

It is notorious that numerous vocal pedagogy practices aim to manage airflow and lung volumes, strongly focused on the concepts of breath support and control. Singing requires greater muscle activity to control the pressures resulting from the increase in lung volume necessary for this activity. This volume is larger than in any other phonatory task. In summary, changes in respiratory muscle strength can change the respiratory support mechanism^(27)^.

Breathing is an important phonation subsystem, responsible for providing the necessary air flow for appropriate subglottic pressure to vibrate the vocal folds^(37).^ The literature points out that the benefits associated with respiratory training improve this subsystem directly, while its effects on the voice remain unclear^(37-39)^. Including respiratory conditioning exercises in a vocal conditioning training program can favor singers, as they improve respiratory awareness and airflow and increase maximum phonation time (MPT).

The articles included in this review mentioned incentive spirometers^(24,25)^ and respiratory muscle strength training devices^(27,29)^. It is not uncommon to have doubts about the use of respiratory incentives and devices in speech-language-hearing clinical practice, although they are increasingly used in clinical practice^(40)^ and in clinical voice research^(31)^. There are incentive spirometers and inspiratory and expiratory devices, with ventilatory dynamics oriented towards flow, volume, and pressure^(39,41)^.

Respiratory muscle strength training utilizes pressure threshold devices, providing specific loads to the respiratory muscles. In contrast, flow-oriented incentive spirometers, such as Respiron^®^, expand lung structures to increase and improve airflow control. However, they do not provide a significant gain in lung volumes or respiratory muscle strength ^(27,41-43)^.

SOVTE were the most frequently used in specific voice training, appearing in four of the eight studies^(24-26,28)^. This modality of voice exercises included humming technique, lip trill, plosive and fricative consonants, and devices such as tubes, straws, and PEP devices. SOVTE involves a partial reduction of the anterior part of the vocal tract (VT) during phonation. This reduction can be produced by articulators, hands, artificial VT stretching with tubes and straws in the air or water, and so forth. Narrowing the VT diameter results in resistance to air exit, which, in turn, modifies the acoustic impedance in the supraglottic region. This process characterizes retroflex resonance (inert reactance), energy that returns to the vocal folds, altering its vibration pattern during SOVTE^(44,45)^. Several positive effects have been attributed to this type of exercise, such as greater source-filter interaction, production of a more resonant voice, and decreased contact coefficient of the vocal folds, reducing the risks of trauma, thus producing the effect called "vocal economy"^(46)^.

Tubes and straws are part of the set of SOVTE and were the most prevalent type of SOVTE in this review. These devices can artificially stretch the TV at low cost and easy access; they can be rigid or flexible, produced in different materials (e.g., glass, silicone, plastic, and metal), and can have different diameters, lengths, and depths of immersion. They can also be used freely in the air or with one end immersed in water^(46,47)^.

A study^(48)^ examined VT and glottal function during and after vocal exercise with resonance tubes and straws, and its findings pointed out several benefits associated with vocal training with these devices. A more resonant voice was perceived, which reflects in a brighter and more intense sonority, a decrease in perceived vocal effort (i.e., an easier and more economical voice), and a slight distancing of the vocal folds, decreasing the impact stress of glottic adduction. Tube phonation can also promote a massage effect when immersed in water, due to bubbling^(36)^. In view of the above, and mainly due to the lower possibility of vocal fold injury, SOVTE was the main intervention strategy for vocal conditioning of singers among the studies investigated.

The parameters for prescribing exercises varied greatly, whereas some studies did not even provide these data. Comparing and synthesizing the findings with such a diversity of information poses a challenge. Standardization could allow for greater reliability, reproducibility, and comparisons between interventions. The most frequent orientation was execution divided and measured in the number of sets and/or repetitions^(24,25,27,30)^. Nonetheless, most studies monitored time either in the total sum of repetitions or in the duration of each repetition. The performances ranged from two^(24,25)^ to five^(27)^ sets with five^(27)^ to 15^(24,25)^ repetitions. Only one publication monitored exercise execution using only time^(29)^.

Recent studies have obtained results different from those of the present review, in which exercise dosage was measured more frequently in terms of time alone^(31,33)^. Another training characteristic to consider is rest time. Only one study^(27)^ recorded and monitored the rest interval between series, ranging from 1 to 3 minutes. There is still no consensus in the literature about rest intervals between sets, but it is known that they influence the recovery of energy sources, lactic acid accumulation, and hormone levels. The higher the training intensity, the longer the rest period needed. Voice rests in general should last 2 to 3 minutes between sets^(1,49)^.

Research on the appropriate dosage of exercise has stood out as an important focus in voice, making it essential to develop criteria that help define the dose and improve the accuracy of vocal prescriptions. According to the science of exercise physiology, neuromuscular exercises should consider each person’s individualities, adjusting parameters such as frequency, duration, intensity, and progression to achieve the desired results. Similarly, vocal conditioning training must consider the order of the exercises, the number of sets, and the rest time^(1,36)^. The results of a study with amateur singers^(50)^ indicated that the exercise with a flexible latex tube immersed in water improved vocal quality from the 3^rd^ to the 5^th^ minute and caused significant negative sensations after the 7^th^ minute. However, this dosage can be differentiated in highly trained singers.

Professionals must carefully analyze the prescription of each exercise, considering the results. Each exercise has specific requirements, which makes it essential to adjust the prescription parameters according to the client's response and the particularities of each exercise. Dose progression is another relevant, necessary aspect; however, it is quite challenging. An adequate management of the variables of conditioning training programs (selection and order of exercises, number of sets and repetitions, duration of rest, and intensity) can ensure that the stimulus-response-adaptation process occurs recurrently until the functional capacity of the muscle tissue increases, not overlooking that an insufficient load may not promote the necessary adaptations for training progress^(49)^. Most publications in this review^(24,25,27,29,30)^ used this training parameter, mainly modifying the selection of exercises, the number of repetitions, and/or the execution time and intensity.

Caution is often recommended about overload, as it can result in fatigue, performance decline, and muscle damage^(51)^. However, adequate planning for vocal conditioning should manage fatigue, which is expected and even desirable in these circumstances. It generates neuronal and metabolic changes that, in turn, help to promote a faster recovery after using the muscles and reduce susceptibility to lesions. That said, an important detail to consider is that conditioning exercises should not be performed on or near days of large presentations, but on days dedicated to training^(6,52)^.

Regarding exercise frequency, almost all publications^(24,25,27-30)^ recommended and monitored daily training, repeating exercises up to 10 times a day, regardless of the frequency of follow-up sessions, which was heterogeneous in this review. In scientific literature on voice, the variables related to the prescription parameters are diverse. However, the physiology of exercise indicates that training repetition is responsible for neuromuscular and metabolic adaptations indispensable for conditioning. Therefore, training focused on vocal improvement should benefit from a regular and long-term exercise program. In this review, the total duration of the interventions ranged from 3^(27)^ to 11 weeks^(24)^. This finding agrees with the literature, which establishes that structural and metabolic changes begin after 4 or 5 weeks on average from the beginning of a conditioning program. The effects of exercises aimed at vocal endurance become noticeable from this period and reach their peak only after about 8 weeks of training^(4).^

The interest in applying the knowledge of exercise physiology in clinical voice has increased in recent decades. In the vocal training of singers, the use of the principle of specificity has increasingly become part of clinical practice. Specificity means that the training must be specific to the activity it intends to improve – i.e., the elements necessary for the actual performance must be part of the conditioning programs as closely as possible^(1,53)^. Thus, although breathing and voice are closely related, training breathing alone does not supply the specificity necessary for vocal conditioning. Other implications concerning singers may be related to their repertoire, for example. Vocal adjustments vary between styles; genres such as Brazilian Popular Music (MPB) have adjustments close to speech, and the larynx has more mobility, while classical singing requires more complex changes in the vocal tract, achieved with years of training, with the larynx being lower and more fixed ^(3,26,54)^.

Professionals who work or wish to work with the vocal conditioning of singers need to be aware of the specific vocal demands of these artists' repertoire, whether they are power, resistance, or both^(3,26,54)^. Training the proposed exercises in different positions or body movements, such as lying down, walking^(27,29)^, or rehearsing an entire presentation with the formal clothes that will be used in a concert^(26)^, were examples of the principle of specificity included in the publications that were part of this review. On the other hand, two studies^(24,25)^ presented training programs for vocal conditioning that were not designed exclusively for singing voices, contemplating spoken voice professionals, such as actors and announcers.

Two studies^(24,25)^ respected the principle of individuality. This is another principle of exercise science related to the person’s characteristics regarding needs, abilities, and capacities. Each person responds differently to stimuli, so it is up to the professional to manage the training, taking this aspect into account. Finally, to maintain the adaptations promoted with a vocal training program, it is necessary to consider the principle of reversibility. Only one study reported observing this principle^(25)^, which determines that the gains obtained with training can be reversed if exercise intensity is reduced (detraining). Moreover, it is more work to restart conditioning than to maintain it^(10)^.

Like the principles of exercise physiology, sports science also uses the principles of motor learning widely. Their purpose is to enhance the acquisition, retention, and generalization of motor behaviors. These concepts aim to encourage awareness for more precise movements, such as self-monitoring and error reduction. An approach based on the principles of motor learning can ensure the proper execution of strategies to condition professional voices, which positively impacts the effects of training and makes singers less vulnerable to lesions^(6,52,55-57)^.

Although it was not the focus of this study, it is important to mention that half of the studies also included indirect approaches^(23,25,26,30)^ to guide participants about vocal hygiene and other aspects. Addressing elements related to vocal health is of great importance to support direct approaches that focus on vocal conditioning. Just like athletes, it is necessary to make singers aware that their performance depends not only on training levels but also on habits that involve a balanced diet, adequate hydration, good hours of sleep, and regular physical exercise. All these interfere with the quality of their performance, fatigue, and recovery levels^(4)^.

Another important aspect was the involvement of professionals with different activities and backgrounds working together in the design and application of training programs. Some studies indicated that the training was conducted by undergraduate or graduate speech-language and audiology students guided by their professor/advisors, alone or in partnership with students from other areas such as physiotherapy^(24,25,30)^, as well as future singing teachers and physical trainers^(28)^ or speech-language-hearing pathologists and singing teachers with extensive experience^(37)^. It is necessary to have a multidisciplinary look at singers, who are multifaceted professionals. They can benefit greatly from the cooperative work of different professionals to achieve their best vocal and artistic performance on stage. It should be noted that this topic still needs to be further explored and disseminated due to its relevance to singers.

Self-evaluation and aerodynamic and acoustic measurements stood out among outcome measures and results. These measures were also part of a study that investigated vocal training in healthy individuals^(36)^. Although it has been the focus of attention for years, there is still no consensus on what would be ideal in evaluating the effects of voice interventions. What is observed is a propensity to use acoustic, aerodynamic, and vocal self-evaluation measures.

Acoustic analysis is an important tool for evaluating the effects of vocal interventions due to its connection with the physiological mechanisms of the larynx, wide clinical application, and nonlinear and multiparametric analyses. Studies show that training healthy voices improves jitter and reduces the noise-to-harmonic ratio (NHR)^(58-60)^. On the other hand, aerodynamic measurements are sensitive enough to capture discrete vocal changes obtained from healthy voice training, since vocal training produces significant effects, such as increased subglottic pressure, decreased transglottic flow, and better aerodynamic efficiency^(31,36,52)^.

However, the current literature highlights that evaluating the direct gains of voice professionals should be the main target. Thus, vocal self-evaluation is a coherent way to monitor these gains, as it can point out the person's sensations and perceptions, directing clinical action towards selecting the best techniques and adjustments in their prescription parameters^(31,36,52)^. Positive self-evaluation results were the most frequent effects of the training programs observed in this review. Participants reported improvements such as increased awareness of breathing control, greater ease in performing certain techniques, greater flexibility, decreased sensation of vocal fatigue, and increased phonatory comfort and motivation^(23-30)^. No study reported negative results.

Patient reported outcome measures (PROMs) stand out among the self-evaluation tools, including the Voice Handicap Index 10 (VHI-10), aimed at measuring the impact of voice disorders on the lives of individuals^(24,61,62)^; the Modern Singing Handicap Index (MSHI), an adaptation of the VHI for singers of modern music, musical theater, and popular music, with items adjusted to their specific needs^(3,63)^; the Voice-Related Quality of Life (VRQOL), which investigates how dysphonia interferes with the quality of life^(3,64)^; the Vocal Fatigue Index (VFI), aimed at identifying vocal fatigue, especially in contexts of prolonged voice use^(24,62,65)^; and the Evaluation of the Ability to Sing Easily (EASE-BR), which evaluates the comfort and ease of vocal emission during singing^(24,62,66)^. Although they are widely used in clinical practice, these PROMs were not developed specifically to assess the self-perception of vocal conditioning in singers, since their main focus is not on this aspect.

To ensure that a measure accurately reflects the health domain for which it is intended, its construct must be well defined, ensuring the validity and reliability of the data obtained^(67,68)^. This characteristic is fundamental in evaluating the effectiveness of vocal conditioning programs. PROMs with well-established constructs can be integrated into clinical practice, aiding in decision-making and monitoring vocal training and the evolution of vocal conditioning. They also allow the acquisition of real-time data, favoring personalized interventions and optimized results^(69)^. The construct validity of a PROM is an essential element for its applicability in different clinical contexts^(69-71)^. Instruments with well-defined constructs enable a more patient-centered care, as they capture relevant information aligned with their needs, contributing to more effective and satisfactory care^(67-71)^.

The authors of the studies included in this review pointed out limitations and/or implications for the future of their research. Among the implications, we can highlight topics that future studies could address, such as the benefits of a more personalized exercise regimen and its effects on vocal parameters^(30)^, the dose and vocal strength requirements for performance with the help of amplification^(26)^, the effects of long-term training, and the effectiveness of respiratory muscle training on acoustic parameters^(29)^.

The limitations found in the studies include equipment that limited the true measurement of intended measurements, the level of experience of the singers, which made it difficult to verify whether the small changes observed were in fact the result of the training program^(27)^, and difficulties inherent to the pandemic, when singers were not performing as before^(24)^. Absence of laryngeal imaging, small sample size, and absence of follow-up evaluations were also pointed out as limitations^(23)^.

The present study raised important data that show a lack of greater accuracy and specificity regarding the characteristics of the research, especially regarding exercise execution. Some limitations can be mentioned, such as the absence of important information on variables related to the samples, the parameters for prescribing the exercises, and the outcome measures, such as heterogeneous methods and variables. Such inaccuracies hinder synthesis, analysis, and generalization of evidence, exposing a weakness in voice research and reinforcing the need for more consistent research in vocal training to support evidence-based clinical practice for this specific population, which is so vulnerable to voice disorders.

## CONCLUSION

There is variability in the intervention strategies for vocal training focused on conditioning singers without vocal complaints. Approaches focusing on simultaneous work between breathing and voice stood out, among which respiratory muscle strength exercises and SOVTE, especially with tube and straw devices, were the most used. Moreover, there was a higher frequency of studies that guided exercise execution by the number of sets and/or repetitions, although most of these also monitored the time. In most cases, the total duration of the intervention reached the minimum time necessary to begin the intended adaptations – i.e., 4 to 5 weeks on average. Self-evaluation was the predominant outcome measure to analyze the results, which were positive in all studies. In addition, the interdisciplinary work between professionals from different areas of activity stood out.

## Appendix A Adaptation of the final search strategy for each database or information source

**Table:**

DATABASE	SEARCH STRATEGY
**PUBMED**	((((((((((((((((((((((((((((((((((((Voice[MeSH Terms]) AND (Singing[MeSH Terms])) OR ("Singing Voice")) OR (Singers)) OR (Singer)) OR ("Healthy Volunteers"[MeSH Terms])) OR ("Healthy Voice") ) AND ("Voice Training"[MeSH Terms]) ) OR ("Vocal Training") ) OR (Exercise[MeSH Terms])) AND (Voice[MeSH Terms]) ) OR ("Vocal Exercises")) OR ("Exercise Physiology")) AND (Voice[MeSH Terms]) ) OR ("Exercise Science")) AND (Voice[MeSH Terms]) )) OR ("Vocal Exercise Physiology") ) OR (Efficiency[MeSH Terms])) AND (Voice[MeSH Terms])) OR ("Muscle Function")) AND (Voice[MeSH Terms])) OR ("Vocal Function")) OR ("Vocal performance")) OR ("Vocal Practice and Performance")) OR ("Vocal efficiency")) OR ("Vocal Economy"))) OR ("Resistance Training"[MeSH Terms]) ) AND (Voice[MeSH Terms]) ) OR ("Strength Training"[MeSH Terms]) ) AND (Voice[MeSH Terms])) OR ("Muscle Fatigue"[MeSH Terms])) AND (Voice[MeSH Terms])
**LILACS/BVS (filter lilacs)**	((Voice) AND (Singing) OR ("Singing Voice") OR (Singers) OR (Singer) OR ("Healthy Volunteers") OR ("Healthy Voice")) AND (("Voice Training") OR ("Vocal Training") OR (Exercise)) AND ((Voice) OR ("Vocal Exercises") OR ("Exercise Physiology")) AND ((Voice) OR ("Exercise Science")) AND ((Voice) OR ("Vocal Exercise Physiology") OR (Efficiency)) AND ((Voice) OR ("Muscle Function")) AND ((Voice) OR ("Vocal Function") OR ("Vocal performance") OR ("Vocal Practice and Performance") OR ("Vocal efficiency") OR ("Vocal Economy") OR ("Resistance Training")) AND ((Voice) OR ("Strength Training")) AND ((Voice) OR ("Muscle Fatigue")) AND (Voice)
**SCOPUS**	((Voice AND Singing) OR ("Singing Voice" OR Singers OR Singer OR "Healthy Volunteers" OR "Healthy Voice")) AND (("Voice Training" OR "Vocal Training") OR (Exercise AND Voice) OR ("Vocal Exercises") OR ("Exercise Physiology" AND Voice) OR ("Exercise Science" AND Voice) OR ("Vocal Exercise Physiology") OR (Efficiency AND Voice) OR ("Muscle Function" AND Voice) OR ("Vocal Function" OR "Vocal performance" OR "Vocal Practice and Performance" OR "Vocal efficiency" OR "Vocal Economy") OR ("Resistance Training" AND Voice) OR ("Strength Training" AND Voice) OR ("Muscle Fatigue" AND Voice))
**EMBASE**	((((((((('voice'/exp OR 'voice') AND ('singing'/exp OR 'singing') OR 'singing voice' OR singers OR 'singer'/exp OR 'singer' OR 'normal human'/exp OR 'normal human' OR 'healthy voice') AND ('voice training'/exp OR 'voice training') OR 'vocal training' OR 'exercise'/exp OR 'exercise') AND ('voice'/exp OR 'voice') OR 'vocal exercises' OR 'exercise physiology'/exp OR 'exercise physiology') AND ('voice'/exp OR 'voice') OR 'exercise science') AND ('voice'/exp OR 'voice') OR 'vocal exercise physiology' OR 'productivity'/exp OR 'productivity') AND ('voice'/exp OR 'voice') OR 'muscle function'/exp OR 'muscle function') AND ('voice'/exp OR 'voice') OR 'vocal function exercise'/exp OR 'vocal function exercise' OR 'vocal performance' OR 'vocal practice and performance' OR 'vocal efficiency' OR 'vocal economy' OR 'resistance training'/exp OR 'resistance training') AND ('voice'/exp OR 'voice') OR 'muscle fatigue'/exp OR 'muscle fatigue') AND ('voice'/exp OR 'voice')
**WEB OF SCIENCE**	ALL=(((Voice) AND (Singing) OR ("Singing Voice") OR (Singers) OR (Singer) OR ("Healthy Volunteers") OR ("Healthy Voice")) AND (("Voice Training") OR ("Vocal Training") OR (Exercise)) AND ((Voice) OR ("Vocal Exercises") OR ("Exercise Physiology")) AND ((Voice) OR ("Exercise Science")) AND ((Voice) OR ("Vocal Exercise Physiology") OR (Efficiency)) AND ((Voice) OR ("Muscle Function")) AND ((Voice) OR ("Vocal Function") OR ("Vocal performance") OR ("Vocal Practice and Performance") OR ("Vocal efficiency") OR ("Vocal Economy") OR ("Resistance Training")) AND ((Voice) OR ("Strength Training")) AND ((Voice) OR ("Muscle Fatigue")) AND (Voice))
**COCHRANE**	(Voice AND Singing OR Singing Voice OR Singers OR Singer OR Healthy Volunteers OR Healthy Voice) AND (Voice Training OR Vocal Training OR Exercise AND Voice OR Vocal Exercises OR Exercise Physiology AND Voice OR Exercise Science AND Voice OR Vocal Exercise Physiology OR Efficiency AND Voice OR Muscle Function AND Voice OR Vocal Function OR Vocal performance OR Vocal Practice and Performance OR Vocal efficiency OR Vocal Economy OR Resistance Training AND Voice OR Strength Training AND Voice OR Muscle Fatigue AND Voice)
**Google Scholar (Top 100)**	Voice AND Singing AND Voice Training OR Exercise Physiology OR Resistance Training OR Strength Training OR Muscle Fatigue
**ProQuest**	Voice AND Singing AND (“Vocal Training” OR "Voice Training")
**MedRxiv**	Voice AND Singing AND “Voice Training”
**Journal of Singing**	Voice AND Singing AND “Voice Training”
**BDTD**	"Singing Voice" AND "Voice Training" OR “Vocal Training”

## Appendix B Data extraction instrument

**Table:**

**Variable**	**Description**
**Year**	Year the article was published
**Authorship**	Names of the authors who conducted the studies
**Country of publication**	Country in which the study was published
**Title**	Assigned name to the job
**Journal**	Journal in which the work was published
**Objective**	Purpose of the study
**Study design**	Type of study classified according to Medronho^(32)^: experimental and quasi-experimental, including randomized clinical trials, non-randomized controlled trials, before and after studies, and case studies and series
**Sample characteristics**	Information on the number of participants, sex and age
**Exercise/technique used**	Description of the interventions that were used to achieve the purpose
**Dosage**	Recommended dose in each study
**Frequency of exercise**	Periodization of training
**Frequency of sessions**	Periodization of follow-up sessions
**Total duration of the intervention**	Total period in which the training lasted
**Professional who applied**	Area of activity of those who applied the intervention or conducted the study
**Outcome measures**	Tests used to evaluate results
**Results**	Effects obtained after the proposed training
**Conclusion**	Final finding that is related to the objective of the study
**Limitations/implications**	Limitations and recommendations brought by the authors of the study
