# Supplementary material for: Mapping the characteristics of training for vocal conditioning of singers: a scoping review
Source: Codas. 2026 Jul 3;38(4):e20250373. doi: 10.1590/2317-1782/e20250373en (PMC13330938; doi:10.1590/2317-1782/e20250373en)
Supplement: Translation

# Translation: Mapeamento das características dos treinamentos para o condicionamento vocal de cantores: uma revisão de escopo

**DOI:** 10.1590/2317-1782/e20250373pt

**Authors:** Pamela Carvalho, Priscila Oliveira, Denis Batista, Flávia Viegas

**Affiliations:** 1 Departamento de Formação Específica em Fonoaudiologia, Universidade Federal Fluminense – UFF - Nova Friburgo (RJ), Brasil.; 2 Departamento de Fonoaudiologia, Universidade Federal da Paraíba – UFPB - João Pessoa (PB), Brasil.; 3 Programa de Pós-graduação em Modelos de Decisão e Saúde, Universidade Federal da Paraíba – UFPB - João Pessoa (PB), Brasil.; 4 Dr Liang Voice Program, The University of Sydney - Sydney (NSW), Australia.

**Keywords:** Canto, Revisão de Escopo, Qualidade da Voz, Treinamento da Voz, Voz

## Abstract

**Objetivo:**

Mapear e caracterizar as estratégias de intervenção para o condicionamento vocal de cantores sem queixa vocal.

**Estratégia de pesquisa:**

Revisão de escopo baseada na pergunta: Como se caracterizam as estratégias de intervenção utilizadas para o condicionamento vocal de cantores? Foram realizadas buscas nas seguintes fontes: LILACS, MEDLINE, Embase, Scopus, Cochrane Library, Web of Science, Google Scholar, medRxiv, ProQuest, Journal of Singing e Biblioteca Digital Brasileira de Teses e Dissertações.

**Critérios de seleção:**

Foram incluídos estudos com treinamento vocal ou respiratório de cantores sem queixas vocais, de qualquer gênero musical, maiores de 18 anos. Excluíram-se estudos com crianças, outros profissionais da voz, participantes com alterações laríngeas e vocais, reabilitação da voz, abordagem exclusivamente indireta ou que não focaram no condicionamento vocal.

**Análise dos dados:**

Os resultados foram sintetizados em tabelas e quadros.

**Resultados:**

Foram identificados 12.358 estudos, oito atenderam aos critérios de elegibilidade. Destacaram-se as abordagens com foco em força muscular respiratória e Exercícios de Trato Vocal Semiocluído. O número de repetições juntamente com monitoramento do tempo total de execução foi o mais utilizado. A duração das intervenções variou entre três semanas e seis meses e a maioria dos programas de treinamento foi conduzida por fonoaudiólogos e profissionais de outras áreas. Houve predominância de autoavaliações, com resultados majoritariamente positivos.

**Conclusão:**

Foi encontrada variabilidade nas estratégias de intervenção utilizadas. O enfoque para um trabalho simultâneo entre respiração e voz se destacou, sendo exercícios de força muscular respiratória e de Trato Vocal Semiocluído as técnicas mais utilizadas.

## INTRODUÇÃO

Cantar adequadamente envolve uma série de fatores, porém, fazê-lo por longos períodos e repetidas vezes exige muito preparo e disciplina do artista^(1)^. O cantor é um profissional da voz diferenciado. Sua atuação profissional envolve alta demanda, especificidade e a necessidade de ótima qualidade da voz. Tais características o posiciona em uma categoria considerada como “elite vocal”^(2)^. A alta demanda vocal expõe o cantor a maior suscetibilidade aos distúrbios vocais, o que ressalta a necessidade de constante atenção e cuidado com o seu instrumento de trabalho. Esse cuidado deve ir além de orientações exclusivamente, uma vez que é necessário um condicionamento específico para que ele possa suportar o uso prolongado da voz^(3)^. Um treino focado na eficiência muscular e na resistência à fadiga é essencial para os artistas que se apresentam por várias horas^(4)^.

O número de apresentações dos profissionais da voz cantada tem aumentado, assim como as exigências em relação ao seu desempenho, que estão se tornando cada vez mais diversificadas^(5)^, portanto eles têm sido comparados aos atletas^(1,4,6,7)^. Seja no esporte ou na arte vocal, é esperado um nível superior de desempenho, ou seja, uma alta *performance*. Tal conceito diz respeito ao sucesso além do esperado, com resultados consistentes e por períodos prolongados, não sendo uma característica inerente ao indivíduo, mas a consequência de um conjunto específico de práticas. Além de manter a estabilidade de um rendimento elevado por longos períodos, quem alcança a alta *performance* também precisa apresentar domínio em muitas das áreas que, de alguma forma, estão relacionadas com essa carreira^(8)^. O condicionamento vocal é uma das competências que o cantor de alta *performance* precisa ter e é por meio dele que este profissional poderá ser capaz de suportar a crescente demanda de apresentações em *shows*, ensaios e gravações, apresentando resultados satisfatórios, com consistência e a longo prazo.

A voz, do mesmo modo que todos os movimentos corporais, é o produto de diversas ações neuromusculares^(9,10)^. Nesse sentido, e principalmente quando exposta ao uso excessivo sem técnica adequada, pode sofrer efeitos negativos, tais quais: falta de resistência, fadiga e esforço para sua produção^(11,12)^. O condicionamento vocal é a preparação dos músculos envolvidos na produção da voz e os seus objetivos incluem: otimizar o desempenho, propiciar resistência à fadiga e favorecer a recuperação muscular, ou seja, sua finalidade é dar condições para que esses músculos sejam capazes de se adaptar às necessidades e exigências impostas aos profissionais da voz falada e cantada, assim, reduzindo os riscos de lesões ou adversidades em sua *performance*. Isso implica em um programa regular de exercícios. É recomendado que um programa de condicionamento seja aplicado em indivíduos vocalmente saudáveis^(4)^. Entretanto, são observadas lacunas na produção científica quanto ao emprego de estratégias de intervenção eficazes para o aumento do desempenho vocal desses artistas.

As revisões de escopo têm se mostrado uma ferramenta útil para o reconhecimento de evidências, podendo ser usadas para fornecer uma visão geral ampla de um tópico^(13)^. Esse tipo de revisão de literatura tem como objetivo mapear os conceitos fundamentais de uma área do conhecimento e permite fazê-lo com desenhos de estudos diversificados, de forma confiável e com qualidade^(14-17)^. O que se pretende alcançar com essa revisão é a identificação e sistematização do conhecimento da área da habilitação vocal, com vistas a apoiar uma melhor atuação por parte dos profissionais que trabalham com o condicionamento vocal de cantores. Com base no exposto, a revisão de escopo se configura como o método mais adequado para essa pesquisa. Desta forma, o objetivo dessa revisão é mapear e caracterizar as estratégias de intervenção disponíveis na literatura científica para o condicionamento vocal de cantores sem queixas vocais.

## MÉTODO

### Delineamento e registro do protocolo

Esta revisão de escopo foi conduzida pela metodologia do *Joanna Briggs Institute* (JBI) para revisões de escopo^(18)^ e descrita conforme o roteiro *Preferred Reporting Items for Systematic Reviews and Meta-Analyses – Extension for Scoping Reviews* (PRISMA-ScR)^(19)^. O protocolo de revisão foi registrado no *Open Science Framework* ^(20)^.

Inicialmente, foi conduzida uma busca preliminar em bases de dados eletrônicas*,* assim como nas plataformas que registram protocolos de revisão em andamento. Nenhuma revisão sistemática ou de escopo com o mesmo objetivo foi identificada.

A presente revisão de escopo foi norteada pela seguinte questão de pesquisa: “Como se caracterizam as estratégias de intervenção utilizadas para o condicionamento vocal de cantores?” A estrutura PCC (população, conceito e contexto) foi utilizada para delinear o estudo, a saber: a população corresponde a cantores sem queixas vocais, de qualquer gênero musical e com idade superior a 18 anos; o conceito está relacionado às características das intervenções realizadas; e o contexto envolve o treinamento, seja ele vocal ou respiratório, com foco no condicionamento.

### Critérios de elegibilidade

Foram considerados os seguintes tipos de estudo: experimentais e quase-experimentais, incluindo ensaios clínicos randomizados, ensaios controlados não randomizados, estudos antes e após intervenção, e estudos de série de casos. Foram excluídos estudos com a população pediátrica, com outros profissionais da voz que não os cantores, com participantes com alterações de laringe e voz, estudos que descreviam intervenções voltadas à reabilitação da voz, com abordagem exclusiva de intervenção indireta ou que não tinham foco no condicionamento vocal. Também não foram considerados estudos observacionais, revisões, cartas ao editor ou editoriais, livros e capítulos de livros, e resumos publicados em anais de eventos científicos.

### Busca e seleção de dados

Uma pesquisa inicial foi conduzida na base de dados PubMed via *MEDLINE* para identificar artigos sobre o tema. As palavras contidas nos títulos e resumos dos artigos relevantes, assim como os termos de indexação usados ​​para descrever os artigos, foram usados ​​para desenvolver uma estratégia de busca inicial, a partir dos elementos do PCC. Essa estratégia de busca foi aprimorada a partir dos primeiros artigos encontrados, considerando todas as palavras-chave e termos de indexação identificados. Foram utilizados os vocabulários de indexação da PubMed (*Medical Subject Headings* – MeSH), Embase (Emtree) e a Biblioteca de Descritores em Ciências da Saúde (DeCS), além de descritores livres baseados no modelo PCC. A estratégia foi adaptada para cada base de dados ou fonte de informação, conforme apresentado no Apêndice A, abrangendo LILACS (BVS), Medline (PubMed), Embase, Scopus, *Cochrane Library* e *Web of Science*. Também foi realizada uma busca manual nas listas de referências dos artigos selecionados a partir das bases de dados eletrônicas, assim como uma pesquisa adicional no *Google Scholar* (100 primeiros estudos), *MedRxiv*, *ProQuest*, Biblioteca Digital Brasileira de Teses e Dissertações (BDTD) e *Journal of Singing*. A inclusão do último periódico se deu devido ao seu elevado impacto específico para a população-alvo do estudo. Não foram aplicadas restrições de idioma ou período a fim de que a sensibilidade desta pesquisa fosse ampliada. As buscas foram realizadas no dia 26 de maio de 2025.

A seleção dos estudos relevantes envolveu duas etapas – triagem de títulos e resumos e leitura completa dos estudos. Antes de dar início à seleção, foi realizada uma calibração dos dois revisores a partir dos primeiros 25 artigos retirados da base de dados *PubMed* (via *MEDLINE*) e o índice de concordância calculado pelo *Cohen’s Kappa Test foi* de 100% (*k*=1). Após a calibração, todas as citações identificadas nas fontes de evidência foram agrupadas e carregadas no Rayyan^(21)^ e as duplicatas removidas. Nas fontes de informação com formato incompatível com o *software* em questão, a busca foi feita de forma manual. Os títulos e resumos foram triados pelos revisores observando os critérios de inclusão para a revisão. Já os textos completos das citações selecionadas foram avaliados detalhadamente em relação aos critérios de exclusão. Todo esse processo foi realizado de forma independente e cega. As divergências entre os revisores foram resolvidas em consenso. Após a inclusão dos artigos por meio de leitura na íntegra, foi realizada uma seleção manual nas listas de referências dos artigos incluídos. Os resultados da pesquisa e do processo de inclusão dos estudos foram relatados e apresentados em um fluxograma (Figura 1).

**Figura 1:** Gráfico de fluxo de trabalho PRISMA^(22)^

Os dados foram extraídos por dois revisores de forma independente e cega, com a utilização de uma planilha de extração de dados previamente desenvolvida para este estudo. Após a extração, os dados foram comparados e as discordâncias foram resolvidas por consenso.

Conforme descrito no Apêndice B as seguintes informações foram coletadas: ano, autoria, país de publicação, título, periódico, objetivo, delineamento de estudo, características da amostra (tamanho amostral, sexo e idade), exercício/técnica utilizada, dosagem, frequência (realização dos exercícios e sessões), duração total da intervenção, profissional que aplicou, medidas de desfecho, resultados, conclusão e limitações/implicações para o futuro.

### Análise e síntese de dados

Os resultados desta revisão foram apresentados por meio de frequências relativas (%) e absolutas (n) e tabelas e quadros em uma síntese descritiva dos achados.

## RESULTADOS

Um total de 12.358 estudos foram identificados a partir das buscas em bases de dados eletrônicas e manuais, sendo removidas 3.066 duplicatas. Dessa forma, 9.292 artigos foram triados com base na leitura dos títulos e resumos, dos quais 9.274 foram excluídos por não atenderem aos critérios de inclusão. Os estudos elegíveis para leitura na íntegra totalizaram 18. Destes, dez foram excluídos devido às seguintes razões: não apresentarem intervenções vocais, os participantes apresentarem queixas ou por abordarem outros assuntos que não o tema desta revisão. Os oito estudos restantes foram considerados elegíveis para esta revisão. O gráfico de fluxo de trabalho PRISMA (Figura 1) mostra a inclusão e exclusão dos artigos selecionados.

Os estudos selecionados para análise foram publicados entre os anos de 1995 e 2024 e o maior número de publicações foi registrado nos últimos quatro anos, com três estudos (37,5%). Dos oito estudos, três foram conduzidos nos Estados Unidos da América (37,5%) , dois no Brasil (25%), um na Alemanha (12,5%), um na Coréia do Sul (12,5%) e um na Turquia (12,5%). Os periódicos com maior número de publicações foram o *Journal of Voice* e Distúrbios da Comunicação, com duas publicações cada (25% cada); *Sprache · Stimme · Gehör, Journal of Singing, Journal of Religion and Health* e *Yonsei Medical Journal* foram responsáveis por uma publicação cada (12,5% cada). Variados desenhos de estudo foram identificados: o delineamento de estudo antes e após intervenção foi o mais prevalente (75%; n = 6); seguido de estudos quase-experimentais (12,5%; n = 1) e experimentais (12,5%; n = 1) (Quadro 1).

**Quadro 1:** Características dos estudos incluídos

**Autor**	**Título**	**Objetivo do Estudo**
Sezin et al.^(23)^	Effectiveness of a Voice Training Program for Religious Officials in Turkey	Examinar a eficácia de um programa de treinamento de voz projetado para autoridades religiosas islâmicas
Ferreira et al.^(24)^	Programa de Condicionamento Vocal e Respiratório (CVR II): nova proposta para profissionais da voz	Descrever um programa de condicionamento vocal e respiratório
Ferreira et al.^(25)^	Programa Condicionamento Vocal e Respiratório (CVR): proposta de intervenção para profissionais da voz	Descrever uma proposta de intervenção que contempla treinamento muscular vocal e respiratório
Sandage e Hoch^(26)^	Training Considerations for Recital Performance: Framing Vocal Dose in a Fatigue Resistance Training Model	Compreender a carga de trabalho necessária para um determinado esforço vocal. Entender como a dose vocal, que quantifica o esforço vocal, pode ser utilizada para guiar o treinamento e a preparação dos cantores, visando reduzir a fadiga percebida durante e após as performances
Ray et al.^(27)^	Effects of Respiratory Muscle Strength Training in Classically Trained Singer	Entender se é possível aumentar a força muscular respiratória com treinamento de limiar progressivo e se o aumento da força muscular respiratória teve efeito mensurável nos resultados da voz
Bengtson-Opitz^(28)^	Vocal Anti-Aging A Fitness-Programme for the Aging Voice	Apresentar um programa de prevenção vocal que busca melhorar a aptidão vocal retardando o envelhecimento da voz em idosos
Nam et al.^(29)^	Specially programmed respiratory muscle training for singers by using respiratory muscle training device (Ultrabreathe®)	Examinar a função pulmonar, a pressão inspiratória máxima (PImáx), a pressão expiratória máxima (PEmáx) e o tempo máximo de fonação (TMF) após treinamento muscular respiratório especialmente programado
Sabol et al.^(30)^	The Value of Vocal Function Exercises in the Practice Regimen of Singers	Investigar os efeitos de exercícios de função vocal isométricos-isotônicos, sobre os parâmetros de produção vocal

O tamanho da amostra variou entre um e 34 participantes. Levando em consideração os estudos que disponibilizaram dados demográficos, o sexo masculino foi predominante, com um total de 43 homens e 25 mulheres e as idades dos participantes variaram entre 20 e 80 anos, com uma média de 23 a 43,7 anos (Tabela 1).

**Tabela 1:** Características das estratégias

**Autor**	**Amostra**	**Exercício/Técnica**	**Dosagem**	**Frequência de Realização dos Exercícios**	**Frequência de Sessões**	**Duração Total da Intervenção**	**Profissional que Aplicou**
Sezin et al.^(23)^	N = 34 Sexo = M Idade = GC - média 44.3 GE - média 43.7	Postura, respiração, aquecimentos vocais, ressonância e projeção	IND	IND	32 sessões ao longo de 8 semanas (8 sessões com abordagem indireta e 24 sessões com abordagem direta)	8 semanas	IND
Ferreira et al.^(24)^	N = 3 (1 cantor) Sexo = M Idade = média 29.6	1. Respiron Athletic 2^®^ 2. Sopro sonorizado no LaxVox^®^ alternando entre modulado, monotom e hiperagudo 3. Sopro sonorizado no New Shaker^®^ alternando entre modulado, monotom, habitual e hiperagudo	1. 2 séries de 10 e 12 repetições 2. 10 repetições com 15 segundos 3. 10 repetições com 6 e 9 segundos	Diariamente 1 vez ao dia	1 encontro por semana com duração de 1h30	11 semanas	2 fonoaudiólogas conduziram a proposta e alunas dos cursos de graduação em fonoaudiologia e fisioterapia assim como suas orientadoras, docentes de cada curso, também acompanharam o processo
Ferreira et al.^(25)^	N = 3 (1 cantor) Sexo = M Idade = IND	1. Respiron Classic^®^ 2. Sopro sonorizado no Canudo de *milk shake* alternando entre monotom, hiperagudo e modulado 3. Sopro sonorizado no Tubo LaxVox^®^ alternando entre monotom, hiperagudo e modulado 4. Sopro sonorizado no Canudo de pirulito alternando entre monotom, hiperagudo e modulado 5. Sopro sonorizado no Canudo *lollipop* alternando entre monotom, hiperagudo e modulado * Exercícios vocais em tempo aproximado entre dois e três minutos	1. 2 séries de 10, 12 e 15 repetições 2. 10 repetições com 12 segundos 3. 10 repetições com 12 e 14 segundos 4. 10 repetições com 14 e 16 segundos 5. 10 repetições com 12 segundos	Diariamente 2 vezes ao dia	1 encontro por semana	8 semanas	O programa foi conduzido por uma fonoaudióloga acompanhada por 2 alunas de graduação em fonoaudiologia e fisioterapia e as alunas foram orientadas por professoras dos seus respectivos cursos
Sandage e Hoch^(26)^	N = 1 Sexo = M Idade = IND	1. Preparação musical com partituras 2. Trabalho técnico: humming, vibração de lábios, fonação em tubos, sílabas neutras e canto com vogais 3. Prática física 4. Ensaio com o acompanhador + prática vocal regular 5. Execução de toda a performance com a roupa formal do concerto	3. 60 minutos por dia (sessões de 20 a 30 minutos)	1. Prática mental + estudo de partitura - 7 dias por semana 3. 5 dias por semana	IND	1. 6 meses 2. e 3. 4 meses 4. e 5. 1 mês	Professora de canto e fonoaudióloga e cantor e professor de canto
				4. Ensaio com o acompanhador + prática vocal regular - 2 dias por semana			
			5. 2 a 3 execuções				
Ray et al.^(27)^	N = 6 Sexo = F Idade = média 28	1. TMI - ajustado a 80% da PImáx 2. TME - ajustado a 80% da PEmáx	5 séries de 5 repetições (intervalos de 1-3 minutos entre cada série)	Diariamente 1 vez ao dia	1 encontro a cada 5 a 10 dias	3 a 7 semanas	IND
Bengtson-Opitz^(28)^	N = 10 Sexo = IND Idade = entre 70 e 80 anos	1. Corpo/ postura/ respiração – despertar a sensibilidade e flexibilidade do músculo reto abdominal e fortalecimento dos intercostais 2. Embocadura/articulação das vogais e consoantes (modelagem do som): a. Mandíbula: vogais abertas b. Lábios: vogais arredondadas e mímicas c. Língua: “exercício da biela” silenciosamente e com as vogais /i/, /a/ d. Palato mole: bocejos, risadas em voz alta e sons de fechamento /k/, /g/, /ng/, /ʃ/ 3. Consoantes – plosivas (vozeadas e desvozeadas) e fluentes (longa ou curta)	10 minutos	Diariamente 3 vezes ao dia	6 encontros ao longo de 6 semanas com duração de 90 minutos	6 encontros	Futuros professores de canto supervisionados pela autora do curso/método e um terapeuta esportivo assume o trabalho com o corpo e a postura
Nam et al.^(29)^	N = 5 Sexo = F Idade = média 23	Variados exercícios com o Ultrabreathe^®^	1. 5 a 10 minutos 2. 60 a 90 minutos 3. aprox. 10 minutos	1 e 3. Diariamente 7 a 10 vezes por dia	IND	Aproximadamente 2 meses	IND
Sabol et al.^(30)^	N = 20 Sexo = 6M e 14F Idade = GE - média 30 GC - média 28	1. Vogal /i/ sustentada em TMF 2. Glissando ascendente com a vogal /o/ 3. Glissando descendente com a vogal /o/ 4. Notas musicais sustentadas em TMF com a vogal /o/ (2 vezes)	2 repetições (15-20 minutos)	Diariamente 2 vezes ao dia	3 vezes por semana	4 semanas	Investigadora principal do estudo e estudantes de pós-graduação em fonoaudiologia que foram treinados na técnica adequada pelo terceiro autor que é um fonoaudiólogo certificado e licenciado com especialização em voz


**Legenda:** M = masculino; F = feminino; GC = grupo controle; GE = grupo experimental; IND = informação não disponível; TMF = tempo máximo de fonação; TMI = treinamento muscular inspiratório; TME = treinamento muscular expiratório; PImáx = pressão inspiratória máxima; PEmáx = pressão expiratória máxima

Dentre os exercícios/técnicas utilizadas destacaram-se as abordagens que tiveram como objetivo a força muscular respiratória, sendo esse tipo de exercício observado em cinco estudos^(24,25,27-29)^, seguido dos Exercícios de Trato Vocal Semiocluído (ETVSO), com quatro estudos^(24-26,28)^. Quatro estudos incluíram estratégias de abordagem indireta com orientações de cuidados vocais^(23,25,26,30)^, três estudos^(23,26,28)^ utilizaram exercícios de ressonância, assim como o *humming* e dois estudos^(26,30)^ utilizaram vogais sustentadas em tom confortável, em glissando ou em melodia musical. Variados dispositivos foram identificados, dentre os quais: faixas elásticas, bolas, canudos, halteres, espelho, rolha, dispositivo de treinamento de força muscular respiratória, tubos, inspirômetros de incentivo e dispositivo de pressão expiratória positiva (PEP) (Tabela 1).

Foi observada variabilidade na dosagem, sendo o método mais utilizado o número de séries e repetições observado em quatro estudos (50%). Três estudos (37,5%) não forneceram detalhes sobre essa variável. A frequência de realização variou de três encontros por semana até períodos maiores, com dez dias de intervalo. Já em relação à duração total da intervenção, esta variou entre três semanas e seis meses. No que diz respeito aos profissionais que aplicaram o treinamento, os mais citados foram os alunos de graduação ou pós-graduação em fonoaudiologia orientados por seus respectivos professores/orientadores que estiveram presentes em 3 estudos (37,5%). Em alguns estudos foi percebida atuação em parceria com estudantes de outras áreas, como os da fisioterapia (Tabela 1).

Sobre as medidas de desfecho, pouco mais da metade dos estudos (n=5; 62,5%) utilizou a autoavaliação vocal. Medidas aerodinâmicas (n=4; 50%), medidas acústicas (n=4; 50%) e medidas de força muscular respiratória (n=3; 37,5%) também foram citadas. Os métodos menos utilizados foram: análise de faixa de frequência vocal (n=2; 25%), análise perceptivo auditiva (n=1; 12,5%), medidas de intensidade (n=1; 12,5%), dose vocal (n=1; 12,5%), função pulmonar (n=1; 12,5%) e videoestroboscopia (n=1; 12,5%) (Quadro 2).

**Quadro 2:** Medidas de desfecho, resultados e conclusão

**Autor**	**Medidas de Desfecho**	**Resultados**	**Conclusão**
Sezin et al.^(23)^	1. Medidas acústicas: f_o_, *jitter, shimmer* e HNR	1. Houve uma redução significativa em *shimmer* (de 1,76% para 1,41%) e *jitter* (de 0,27% para 0,21%). A HNR aumentou significativamente (de 25,44 para 26,76). Não houve variação significativa na f_o_	Observou-se resultados benéficos de um programa de educação e treinamento em voz projetado para funcionários religiosos islâmicos
	2. Autoavaliação: IDV-C, IFV e QVV	2. IDV-C e IFV - foi relatada uma diminuição significativa na percepção de desvantagem vocal (de 18,00 para 12,17) e nos sintomas de fadiga vocal relatados (de 29,05 para 19,82). QVV - A qualidade de vida relacionada à voz melhorou significativamente (de 68,00 para 86,76). O GC não apresentou mudanças significativas em nenhum dos parâmetros avaliados, indicando que as melhorias observadas no GE foram atribuídas ao programa de treinamento vocal	
Ferreira et al.^(24)^	1. Análise perceptivo-auditiva: vogal /a/ emitida 3 vezes e fala espontânea - *pitch, loudness*, ressonância, coordenação pneumofonoarticulatória, velocidade de fala, articulação e modulação	1. Aumentou, diminuiu ou estabilizou	O uso de incentivadores respiratórios como proposta para melhorar o condicionamento vocal e respiratório e a parceria com a fisioterapia são apresentados e recomendados para melhor entendimento e consequente atendimento das questões da voz e da respiração em profissionais da voz
	2. Medidas aerodinâmicas: TMF	2. Aumentou	
	3. Autoavaliação: perguntas dirigidas, EAV, IFV, IDV-10 e EASE-BR	3. Maior projeção vocal, melhor possibilidade de produzir sons mais agudos e mais flexibilidade vocal. IFV - diminuição da fadiga e restrição vocal para 2 participantes e desconforto vocal para 1 deles, IDV-10 - mesmo antes não houve desvantagem. Registro ainda mais inferior para 2 participantes após o treinamento e EASE-BR - escores das subescalas variaram entre os participantes	
	4. Medidas respiratórias: força muscular respiratória (PImáx e PEmáx) e VVM	4. Considerável melhora nos índices respiratórios e diminuição na percepção do esforço respiratório	
Ferreira et al.^(25)^	1. Autoavaliação: perguntas dirigidas, sistema de registro de notas de 0 a 10 referente à qualidade da voz e conforto fonatório, IDV e EASE-BR	1. Foram relatadas melhora na autopercepção, motivação, controle vocal e respiratório e no conforto fonatório. Houve diminuição na sensação de fadiga. Observou-se que a melhora da respiração não trouxe repercussão imediata na voz	A proposta mostrou-se viável e promissora, principalmente quando realizada de forma contínua e com progressão gradual dos exercícios. A integração das áreas de fonoaudiologia e fisioterapia é benéfica para otimizar o condicionamento vocal e respiratório. O Programa apresentado tem potencial para registro de efeitos positivos
Sandage e Hoch^(26)^	1. Autoavaliação: IFV e PPE	1. IFV - as medidas foram maiores após cada tentativa de canto em comparação com a medição pré-canto; no entanto, caíram entre os ensaios conforme hipotetizado, PPE - não mudou mais do que alguns pontos nos quatro ensaios	A menor fadiga vocal percebida ao longo dos ensaios para o recital fornece evidências preliminares de um efeito de treinamento de condicionamento para atender às demandas de desempenho deste repertório especificamente e de recital em geral
	2. Medidas de dose vocal	2. Dose vocal - foi ambígua para os dois momentos medidos, estabelecendo uma dose vocal consistente nos dois últimos ensaios (2954,56 metros durante os ensaios e 3098,18 metros durante o recital, com uma intensidade média de som de 93 dB e 94 dB)	
Ray et al.^(27)^	1. Medidas respiratórias: PImáx e PEmáx	1. Houve aumento significativo nas medidas de PImáx e PEmáx. (TMI (fase B) PImáx 37%, 101% e 118% / PEmáx 23%, 45% e 130% - TME (fase C) PEmáx 70%, 28% e 137% / PImáx 16%, 33% e 34% - TMI e TME (fase D) PImáx 63% / PEmáx 104%)	Os resultados mostraram que os cantores aumentaram a força muscular respiratória, tanto na forma de treinamento inspiratório (TMI) quanto expiratório (TME), após a conclusão do programa de treinamento. Não houve mudanças consistentes nas medidas de aerodinâmica e voz entre os sujeitos, embora algumas mudanças individuais tenham sido observadas
	2. Medidas acústicas: Fonetograma: faixa de f_o_, faixa de intensidade (NPS) e fluxo de ar para cada tom	2. Faixa de f_o_ - 3 participantes apresentaram alcance de tom aumentado. Nenhum dos participantes diminuiu a amplitude do *pitch* durante o treinamento. Faixa de Intensidade - foram registrados tanto aumento quanto diminuição no NPS. Fluxo de ar - as medidas foram inconsistentes	
Bengtson-Opitz^(28)^	IND	Todos os participantes experimentaram uma melhora claramente audível e perceptível com melhor articulação, flexibilidade e alcance do tom	O "*Anti-Aging* para a Voz" não é uma solução mágica, mas sim um processo contínuo que requer prática e dedicação. A manutenção dos exercícios e técnicas aprendidas é fundamental para sustentar os benefícios a longo prazo
Nam et al.^(29)^	1. Medidas aerodinâmicas: TMF	1. Aumentou significativamente (de 22.3 ± 4.3seg. para 31.0 ± 6.7seg)	O treinamento muscular respiratório especialmente programado pode melhorar a força muscular respiratória e a função vocal sem aumentar a função pulmonar. Esse método pode ser realizado facilmente e não requer muito tempo
	2. Medidas respiratórias: testes de função pulmonar (curva de fluxo volume, pletismografia - CVF, VEF no 1º segundo, VEF1/CVF e FEF 25 - 75%, CPT e CV e teste de força muscular respiratória (PImáx e PEmáx)	2. Testes de função pulmonar - mostraram-se estatisticamente semelhantes antes e após o treinamento. Força muscular respiratória - apresentaram aumento estatisticamente significativo (PImáx de 62.2 ± 13.5 cmH_2_O para 78.2 ± 3.7cmH_2_O / PEmáx de 56.4 ± 7.5cmH_2_O para 70.0 ± 8.1cmH_2_O )	
	
Sabol et al.^(30)^	1. Medidas acústicas: f_o_, *jitter* e faixa de frequência	1. f_o_ - Não houve diferenças significativas. *Jitter* - A taxa média aumentou em tom confortável (de 0.24 para 0.31Hz) e baixo (de 0.26 para 0.32Hz). Faixa de frequência - Não houve diferenças significativas entre o grupo controle e experimental	Exercícios de função vocal contendo o princípio isométrico-isotônico demonstraram ter um efeito positivo nos sistemas fonatórios de jovens cantores saudáveis. Os resultados indicam que a prática regular desses exercícios pode levar a um aumento na eficiência glótica
	2. Medidas aerodinâmicas: TMF, volume de fonação e taxa de fluxo	2. TMF - Houve aumento no grupo experimental (9s tom confortável, 11s tom agudo e 9s tom baixo). Volume de fonação - o grupo experimental teve um ligeiro aumento (de 2,739 para 2,099ml) . Taxa de fluxo - o grupo experimental teve suas taxas de fluxo diminuídas (de 205 para 133ml/s)	
	3. Videoestroboscopia	3. Não houve diferenças significativas entre o pré e pós-teste	
	4. Autoavaliação: escritas sobre os efeitos dos exercícios de função vocal em seu desempenho de canto	4. Os participantes relataram um aumento na consciência do controle e relaxamento da respiração e melhorias na execução de glissandos. Relataram também maior consciência de tensões laríngeas e faciais. Os três homens do grupo experimental reclamaram de sensações tensas, desconfortáveis ​​e apertadas, especialmente na primeira semana. No entanto, eles melhoraram seus tempos máximos de fonação ao longo das semanas seguintes	

**Legenda:** f_o_ = frequência fundamental; *jitter* = porcentagem de perturbação da frequência vocal; *shimmer* = porcentagem de perturbação da intensidade vocal; HNR = relação harmônico-ruído; IDV-C = Índice de Desvantagem Vocal para o Canto; IFV = Índice de Fadiga Vocal; QVV = Qualidade de Vida em Voz; GC = grupo controle; GE = grupo experimental; TMF = tempo máximo de fonação; EAV = escala analógico-visual; IDV-10 = Índice de Desvantagem Vocal 10; EASE-BR = *Evaluation of The Ability to Sing Easily*; PImáx = pressão inspiratória máxima; PEmáx = pressão expiratória máxima; VVM = *endurance* muscular respiratória; IDV = Índice de Desvantagem Vocal; PPE = escala visual analógica de esforço fonatório percebido; NPS = nível de pressão sonora; TMI = treinamento muscular inspiratório; TME = treinamento muscular expiratório; CVF = capacidade vital forçada; VEF = volume expiratório forçado; FEF = fluxo expiratório forçado; CPT = capacidade pulmonar total; CV = capacidade vital

Em relação aos resultados obtidos com os programas de treinamento, os efeitos positivos na autoavaliação foram mais frequentes (n=5; 62,5%). Não houve diferença na videolaringoestroboscopia nem em medidas de intensidade e frequência (n=1; 12,5%). Nenhum estudo relatou resultados negativos (Quadro 2). A maioria dos estudos apontou limitações e/ou implicações para o futuro em suas pesquisas (n=7; 87,5%). Como por exemplo os benefícios de um regime de exercícios mais personalizado e seus efeitos nos parâmetros vocais^(30)^ (n=1; 12,5%) ou os efeitos de um treinamento a longo prazo e a eficácia do treinamento muscular respiratório nos parâmetros acústicos^(29)^ (n=1; 12,5%) que foram apontados como implicações para o futuro. Enquanto nas limitações foram citados: equipamentos que limitaram a verdadeira mensuração de medidas pretendidas^(27)^ (n=1; 12,5%), dificuldades inerentes ao momento da pandemia quando os cantores não estavam atuando da mesma forma como antes^(24)^ (n=1; 12,5%), ausência de imagem laríngea, tamanho de amostra reduzido e ausência de avaliações de acompanhamento^(23)^ (n=1; 12,5%).

## DISCUSSÃO

Esta revisão mapeou e caracterizou as estratégias de intervenção voltadas ao treinamento vocal para o condicionamento de cantores sem queixas vocais disponíveis na literatura científica. Os resultados podem contribuir para subsidiar a atuação de profissionais da fonoaudiologia que trabalham com cantores, a fim de garantir um comportamento vocal mais efetivo para estes profissionais.

Foram identificados oito estudos^(23-30)^ sobre o condicionamento vocal de cantores publicados entre 1995 e 2024. O número reduzido de estudos encontrados nesta revisão evidencia que pouco se tem discutido sobre essa perspectiva da atuação fonoaudiológica, na área de voz, nas últimas décadas. Os estudos publicados nos últimos quatro anos^(23-25)^ foram mais frequentes, demonstrando que esse tipo de atuação pode estar ganhando visibilidade no campo científico vocal.

A maioria dos estudos utilizou um desenho antes e após^(24-29)^, dado semelhante a outra revisão que mapeou dispositivos usados em terapia e treinamento vocal^(31)^. Os estudos que empregam este tipo de desenho buscam verificar os efeitos da intervenção em um único grupo de participantes, entretanto, apresentam limitações metodológicas, tais quais a falta de grupo controle e randomização dos participantes. Apenas uma fonte de informação^(23)^ utilizou o desenho experimental que é tido como o mais apropriado para determinar se uma intervenção foi eficaz^(32)^.

As amostras dos estudos foram compostas por ambos os sexos, sendo a maioria do sexo masculino com uma média de 23 a 43.7 anos. Dentre os estudos que disponibilizaram os dados demográficos - variável idade, um estudo^(28)^ informou que os participantes tinham entre 70 e 80 anos, porém não informou mais detalhes a esse respeito. Ressalta-se a necessidade de investigações científicas que analisem os efeitos dos programas de condicionamento vocal em diferentes ciclos de vida e gêneros^(33)^.

Os grupos de intervenção variaram entre um e 34 participantes. Em investigações científicas, amostras reduzidas podem dificultar níveis adequados de significância e poder estatístico, impossibilitando que os resultados sejam generalizados para uma população maior. Dessa forma, entende-se que a realização de cálculos amostrais possa dirimir essa questão metodológica^(34)^.

Foi observada variabilidade no que tange aos objetivos das pesquisas. Estas propuseram investigar efeitos dos exercícios^(29,30)^; entender se é possível aumentar a força muscular respiratória e verificar se, ao fazê-lo, houve repercussão na voz^(27)^; compreender como a dose vocal pode ser utilizada para guiar o treinamento(25); examinar a eficácia de um programa de treinamento^(23)^ e, por fim, destacaram-se três estudos que apresentaram programas de treinamento com foco específico no condicionamento da voz^(24,25,28)^. Há escassez na literatura de fontes de pesquisa que retratam programas de condicionamento vocal, sendo que este fato poderia ser creditado a algumas razões, como, por exemplo, ao recente interesse pela atuação com o treinamento vocal de cantores saudáveis e ao conhecimento restrito sobre aspectos bioenergéticos e respostas ao treinamento por parte da musculatura laríngea e do mecanismo de produção vocal.

Metade dos estudos fez uso de estratégias voltadas para uma abordagem conjunta entre as funções respiratórias e vocal^(23-25,28)^, como também foram encontrados estudos que abordaram isoladamente o treinamento da voz^(26,30)^ ou da respiração^(27,29).^ De forma similar ao encontrado na metade dos estudos presentes nessa revisão, um estudo^(35)^, ao apresentar um programa de condicionamento, trouxe um trabalho com proporção igual para exercícios respiratórios e de voz – no entanto, os efeitos deste programa ainda não foram investigados. Neste ponto, é relevante salientar que, quando se trata do trabalho com condicionamento vocal, é difícil estabelecer na literatura objetivos claros, pois ainda são muitas as dúvidas e indefinições conceituais nesse contexto.

Os exercícios de condicionamento respiratório foram predominantes, sendo relatados em cinco dos oito estudos^(24,25,27-29)^. Este resultado contrasta com uma pesquisa^(36)^ em que a abordagem mais utilizada no treinamento de indivíduos com vozes saudáveis foi uma estratégia focada na voz - os Exercícios de Trato Vocal Semiocluído (ETVSO), principalmente com fonação em tubos e canudos. Este achado demonstra uma tendência que pode estar emergindo a partir de um maior entendimento sobre as particularidades do treinamento do cantor.

É notório que inúmeras práticas de pedagogia vocal estão direcionadas ao gerenciamento do fluxo de ar e dos volumes pulmonares, com forte centralização nos conceitos de suporte e controle da respiração. Cantar demanda maior atividade muscular para controlar as pressões resultantes do aumento de volume pulmonar necessário para essa atividade. Tal volume é mais amplo do que em qualquer outra tarefa fonatória. Em síntese, alterações na força muscular respiratória podem resultar em alterações no mecanismo de suporte respiratório^(27)^.

A respiração é um importante subsistema da fonação, responsável por proporcionar o fluxo de ar necessário para que ocorra pressão subglótica apropriada para vibrar as pregas vocais^(37)^. A literatura aponta que os benefícios associados ao treino respiratório trazem melhorias direcionadas a este subsistema, enquanto seus efeitos na voz ainda permanecem pouco claros^(37-39)^. Incluir exercícios de condicionamento respiratório em um programa de treinamento para o condicionamento vocal pode ser favorável aos cantores visto que, aprimoram a consciência respiratória, o fluxo de ar e aumentam o TMF (tempo máximo de fonação).

Nos artigos incluídos nesta revisão, foram citados inspirômetros de incentivo^(24,25)^ assim como dispositivos de treinamento de força muscular respiratória^(27,29)^. Não é incomum a dúvida sobre a utilização de incentivadores e dispositivos respiratórios na clínica fonoaudiológica, embora haja uma crescente atuação com a utilização desses recursos tanto na prática clínica^(40)^ quanto nas pesquisas em voz clínica^(31)^. Existem incentivadores e dispositivos de ação inspiratória e expiratória, com dinâmica ventilatória orientada a fluxo, volume ou pressão^(39,41)^.

O treinamento da força muscular respiratória utiliza dispositivos com limiar de pressão, proporcionando cargas específicas aos músculos respiratórios. Já os incentivadores que são orientados a fluxo, como o Respiron^®^, promovem a expansão das estruturas pulmonares, aumento e melhor controle do fluxo de ar. No entanto, não proporcionam um ganho significativo nos volumes pulmonares ou na força da musculatura respiratória ^(27,41-43)^.

No âmbito do treinamento específico da voz, os Exercícios de Trato Vocal Semiocluído (ETVSO) foram os mais frequentemente utilizados, aparecendo em quatro dos oito estudos^(24-26,28)^. Dentro dessa modalidade de exercícios de voz estiveram presentes as técnicas de *humming*, vibração de lábios, consoantes plosivas e fricativas e dispositivos como tubos, canudos e o dispositivo de pressão expiratória positiva (PEP). Os ETVSO envolvem uma redução parcial da parte anterior do trato vocal (TV) durante a fonação. Essa redução pode ser produzida pelos articuladores, pela mão, pelo alongamento artificial do TV com tubos e canudos no ar ou na água, dentre outros. O estreitamento do diâmetro do TV traz como consequência uma resistência à saída de ar que, por sua vez, modifica a impedância acústica na região supraglótica. Esse processo caracteriza a ressonância retroflexa (reatância inertiva), energia que retorna às pregas vocais alterando o seu padrão de vibração durante a realização dos ETVSO^(44,45)^. Vários efeitos positivos têm sido atribuídos a esse tipo de exercício, como maior interação fonte-filtro, produção de voz mais ressonante e diminuição do coeficiente de contato das pregas vocais, reduzindo os riscos de trauma, assim, produzindo o efeito denominado “economia vocal”^(46)^.

Tubos e canudos fazem parte do conjunto de ETVSO e, dentro desse grupo, representaram o tipo de ETVSO mais prevalente nessa revisão. Estes dispositivos são capazes de alongar artificialmente o TV, possuem baixo custo e são de fácil acesso, podem ser rígidos ou flexíveis, produzidos em materiais distintos, tais quais, vidro, silicone, plástico, metal, assim como podem ter diferentes diâmetros, comprimentos e profundidades de imersão. Além de tudo, podem ser utilizados livres no ar ou com uma das extremidades imersa em água^(46,47)^.

Um trabalho^(48)^ examinou o TV e a função glótica durante e após exercício vocal com tubo de ressonância e canudo e seus achados apontaram diversos benefícios associados ao treinamento vocal com estes dispositivos. Foi percebida uma voz mais ressonante, o que reflete em uma sonoridade com maior brilho e intensidade; uma diminuição no esforço vocal percebido, ou seja, uma voz mais fácil e econômica e, também, um leve afastamento das pregas vocais, o que leva a uma diminuição no estresse de impacto da adução glótica. A fonação em tubos também pode promover um efeito de massagem quando imerso em água, pelo borbulhamento^(36)^. Diante do exposto e, principalmente, devido à menor possibilidade de lesão nas pregas vocais, os ETVSO têm sido a principal escolha de estratégia de intervenção para o condicionamento vocal de cantores, dentre os estudos investigados.

Com relação aos parâmetros de prescrição dos exercícios houve grande variabilidade e, ao mesmo tempo, dados inexistentes em alguns estudos. Comparar e sintetizar os resultados dos achados na presença de tamanha diversidade das informações torna-se um desafio. A padronização poderia permitir maior confiabilidade, reprodutibilidade e comparações entre intervenções. A orientação de execução dividida e medida em número de séries e/ou número de repetições foi a mais frequente^(24,25,27,30)^, entretanto, cabe ressaltar que, na maioria dos estudos citados, o tempo também foi monitorado, seja no somatório total das repetições ou na duração de cada repetição. As execuções variaram de duas^(24,25)^ a cinco^(27)^ séries com cinco^(27)^ a 15^(24,25)^ repetições. Somente uma publicação monitorou a execução dos exercícios utilizando unicamente o tempo^(29)^.

Pesquisas recentes obtiveram resultados distintos da presente revisão, nas quais a dosagem dos exercícios foi medida, com maior frequência, em termos de tempo, exclusivamente^(31,33)^. Outra característica do treino a ser considerada é o tempo de descanso. Foi observado que apenas um estudo^(27)^ registrou e monitorou o intervalo de descanso entre cada série, sendo de um a três minutos. Ainda não há consenso na literatura sobre os intervalos de descanso entre as séries, mas sabe-se que estes influenciam a recuperação das fontes de energia, o acúmulo de ácido lático e os níveis hormonais. Quanto maior a intensidade do treinamento, maior o período de descanso necessário. Na área de voz, de uma forma geral, deve-se descansar de dois a três minutos entre as séries^(1,49)^.

A investigação sobre a dosagem apropriada de exercícios tem se destacado como um foco importante na área da voz, tornando essencial o desenvolvimento de critérios que auxiliem na definição da dose e na melhoria da precisão das prescrições vocais. De acordo com a ciência da fisiologia do exercício, ao prescrever exercícios neuromusculares, devem ser consideradas as individualidades de cada indivíduo, ajustando parâmetros como frequência, duração, intensidade e progressão para alcançar os resultados desejados. De maneira semelhante, no treinamento para o condicionamento vocal, é essencial considerar a ordem dos exercícios, a quantidade de séries e o tempo de descanso^(1,36)^. Em estudo realizado com cantores amadores^(50)^, os resultados apontaram que o exercício com tubo flexível de látex imerso em água, promoveu melhora na qualidade vocal do terceiro até o quinto minuto, e causaram sensações negativas significativas após o sétimo minuto. Contudo, essa dosagem pode ser diferenciada em cantores altamente treinados.

É fundamental que o profissional analise com atenção a prescrição de cada exercício, levando em conta os resultados obtidos. Cada exercício possui exigências específicas, o que torna indispensável ajustar os parâmetros de prescrição de acordo com a resposta do cliente e as particularidades de cada exercício. De forma complementar, outro relevante aspecto a ser mencionado é o uso da progressão da dose. Esse aspecto é tido como necessário, porém, mostra-se bastante desafiador. Uma gestão adequada das variáveis do programa de treinamento para o condicionamento (seleção e ordem dos exercícios, número de séries e de repetições, duração do período de descanso e intensidade) pode garantir que o processo de estímulo-resposta-adaptação ocorra de forma recorrente até que haja um aumento da capacidade funcional do tecido muscular, sem perder de vista que uma carga insuficiente pode não promover as adaptações necessárias para a evolução do treinamento^(49)^. A maioria das publicações presentes nesta revisão^(24,25,27,29,30)^ fez uso deste parâmetro de treinamento, modificando, principalmente, a seleção dos exercícios, o número de repetições e/ou o tempo de execução e a intensidade.

Frequentemente é recomendada cautela no que se refere a sobrecarga, já que essa pode resultar em fadiga, declínio na *performance*, e danos musculares^(51)^. Todavia, um planejamento adequado ao condicionamento vocal deve contemplar o gerenciamento da fadiga, sendo a fadiga, nestas circunstâncias, esperada e até desejável, pois gera mudanças neuronais e metabólicas que, por sua vez, auxiliam na promoção de uma recuperação mais rápida no pós-uso da musculatura além de diminuir a suscetibilidade a lesões. Dito isso, um detalhe importante a ser considerado é o fato de que os exercícios de condicionamento não devem ser realizados em dias ou próximos de dias de grandes apresentações, mas sim em dias dedicados ao treinamento^(6,52)^.

Quanto à frequência de realização dos exercícios, foi percebido que, independentemente da frequência de sessões de acompanhamento, que se mostrou heterogênea nesta revisão, o treino diário foi recomendado e monitorado em quase todas as publicações^(24,25,27-30)^, com a repetição do treino podendo ocorrer até dez vezes ao dia. Na literatura científica de voz, as variáveis relacionadas aos parâmetros de prescrição são diversas, porém, na fisiologia do exercício, preconiza-se que a repetição do treino é responsável por adaptações neuromusculares e metabólicas indispensáveis para o condicionamento. Logo, o treinamento focado no aperfeiçoamento vocal deve se beneficiar de um programa de exercícios regular e a longo prazo. Na presente revisão a duração total das intervenções variou de, no mínimo, três^(27)^ a, no máximo, onze semanas^(24)^. Este achado está de acordo com a literatura, que estabelece que as alterações estruturais e metabólicas começam a ocorrer, em média, após quatro ou cinco semanas do início de um programa de condicionamento. Os efeitos dos exercícios voltados para a resistência vocal tornam-se perceptíveis a partir desse período e atingem seu auge somente após cerca de oito semanas de treinamento^(4)^.

Nas últimas décadas, o interesse em aplicar os conhecimentos da fisiologia do exercício na clínica vocal tem aumentado. No treinamento vocal de cantores, o uso do princípio da especificidade tem feito, cada vez mais, parte da atuação clínica. Especificidade significa que o treinamento deve ser específico para a atividade que se pretende aprimorar, ou seja, os elementos necessários para o desempenho real devem fazer parte dos programas de condicionamento de forma mais aproximada possível^(1,53)^. Desse modo, é possível constatar que, embora haja estreita relação entre respiração e voz, o treino isolado da respiração não supre a especificidade necessária ao condicionamento vocal. Outras implicações em relação aos cantores podem estar relacionadas ao repertório, por exemplo. Os ajustes vocais variam entre estilos: gêneros como a Música Popular Brasileira (MPB) têm ajustes próximos à fala e a laringe possui mais mobilidade, enquanto o canto clássico exige mudanças mais complexas no trato vocal, alcançadas com anos de treino, estando a laringe mais baixa e fixa ^(3,26,54)^.

Os profissionais que atuam ou desejam atuar com o condicionamento vocal de cantores precisam estar atentos às demandas vocais específicas do repertório destes artistas, sejam elas de potência, resistência, ou ainda, de ambas^(3,26,54)^. Treinar os exercícios propostos em diferentes posições ou movimentos corporais, como deitado, caminhando^(27,29)^ ou, ensaiar toda a *performance* de uma apresentação com a roupa formal que será usada em um concerto^(26)^ foram exemplos de inclusão do princípio da especificidade encontrados nas publicações que fizeram parte desta revisão. Em contrapartida, dois estudos^(24,25)^ apresentaram programas de treinamento para o condicionamento vocal que não foram pensados, exclusivamente, para o uso da voz cantada, contemplando profissionais da voz falada, como atores e locutores.

O respeito ao princípio da individualidade foi identificado em dois estudos^(24,25)^. Esse é mais um princípio da ciência do exercício e está relacionado às características próprias que o indivíduo traz quanto às suas necessidades, habilidades e capacidades. Cada pessoa responde de um modo diferente a estímulos, dessa forma, cabe ao profissional gerenciar o treinamento levando este aspecto em consideração. Por fim, com a intenção de manter as adaptações promovidas com um programa de treinamento vocal, é preciso considerar o princípio da reversibilidade. Apenas um estudo relatou observar esse princípio^(25)^, que determina o seguinte: os ganhos obtidos com os treinamentos podem ser revertidos caso haja redução da intensidade do exercício (destreino). Ademais, é mais trabalhoso reiniciar o condicionamento que mantê-lo^(10)^.

Assim como os princípios da fisiologia do exercício, os princípios da aprendizagem motora também são amplamente utilizados pela ciência do esporte. O seu propósito está em potencializar a aquisição, retenção e generalização de comportamentos motores. Estes conceitos estão voltados a incentivar a conscientização para uma produção de movimentos com maior precisão, tal qual o automonitoramento e a redução do erro. Uma abordagem fundamentada nos princípios da aprendizagem motora pode garantir a execução adequada das estratégias voltadas ao condicionamento de vozes profissionais, o que impacta positivamente nos efeitos do treino e torna o cantor menos vulnerável a lesões^(6,52,55-57)^.

Embora não tenha sido foco deste trabalho, é importante citar que metade dos estudos contemplou, também, abordagens indiretas^(23,25,26,30)^ com a intenção de orientar os participantes a respeito de higiene vocal, dentre outros. Abordar elementos relacionados à saúde vocal tem grande importância para dar apoio às abordagens diretas que tem foco no condicionamento vocal. Assim como os atletas, é preciso conscientizar o cantor de que seu desempenho não depende apenas de seus níveis de treinamento, mas também de seus hábitos que precisam envolver dieta equilibrada, hidratação adequada, boas horas de sono e exercícios físicos regulares. Tudo isso interfere na qualidade de sua atuação, nos níveis de fadiga e de recuperação^(4)^.

Outro aspecto importante foi observado: o envolvimento de profissionais com diferentes atuações e formações trabalhando em conjunto na concepção e aplicação dos programas de treinamento. Alguns estudos apontaram que a condução dos treinos foi realizada por alunos de graduação ou pós-graduação em fonoaudiologia orientados por seus respectivos professores/orientadores, sozinhos ou em parceria com estudantes de outras áreas como os da fisioterapia^(24,25,30)^, bem como futuros professores de canto e treinadores físicos^(28)^ ou fonoaudiólogos e professores de canto com muita experiência^(37)^. Faz-se necessário um olhar multidisciplinar para o cantor, que é um profissional multifacetado. Pode ser muito benéfico que diferentes profissionais possam atuar em conjunto para que esse artista chegue em sua melhor *performance* vocal e artística ao palco. Salienta-se que essa temática ainda precisa ser mais explorada e difundida em virtude de sua relevância para o cantor.

No que concerne às medidas de desfecho e seus resultados, destacaram-se a autoavaliação, as medidas aerodinâmicas e as medidas acústicas. De forma semelhante, estas medidas também fizeram parte de estudo que investigou o treinamento vocal em indivíduos saudáveis^(36)^. Embora seja o foco de atenção ao longo de anos, ainda não existe um consenso sobre o que seria ideal na avaliação dos efeitos das intervenções vocais. O que se observa é uma propensão ao uso de medidas acústicas, aerodinâmicas e de autoavaliação vocal.

A análise acústica se constitui uma ferramenta importante para avaliação dos efeitos das intervenções vocais devido à sua conexão com os mecanismos fisiológicos da laringe, ampla aplicação clínica e análises não lineares e multiparamétricas. Estudos mostram que, em vozes saudáveis, o treinamento vocal melhora o *jitter* e reduz a proporção ruído-harmônico (NHR)^(58-60)^. Já as medidas aerodinâmicas possuem sensibilidade suficiente para captar discretas mudanças vocais obtidas no treinamento de vozes saudáveis, visto que, a partir do treinamento vocal, são observados efeitos significativos como aumento da pressão subglótica, diminuição do fluxo transglótico e melhor eficiência aerodinâmica^(31,36,52)^.

Por outro lado, a literatura atual destaca que avaliar os ganhos diretos dos profissionais da voz deve ser o principal alvo. Assim, a autoavaliação vocal desponta como uma forma coerente de monitorar estes ganhos, pois ela é capaz de apontar as sensações e percepções do indivíduo, direcionando a atuação clínica no que diz respeito à seleção das melhores técnicas e aos ajustes em seus parâmetros de prescrição ^(31,36,52)^. Em relação aos efeitos obtidos com os programas de treinamento que fizeram parte desta revisão, os resultados positivos na autoavaliação foram mais frequentes. Os participantes relataram melhorias como aumento na consciência do controle da respiração, maior facilidade na execução de determinadas técnicas, maior flexibilidade, diminuição na sensação de fadiga vocal, assim como aumento do conforto fonatório e da motivação^(23-30)^. Nenhum estudo relatou resultados negativos.

Entre as ferramentas de autoavaliação, destacam-se os *Patient Reported Outcome Measures* (PROMs), que incluem: o Índice de Desvantagem Vocal 10 (IDV-10), voltado para mensurar o impacto das alterações vocais na vida dos indivíduos^(24,61,62)^; o Índice de Desvantagem para o Canto Moderno (IDCM), uma adaptação do IDV direcionada a cantores do canto moderno, teatro musical ou canto popular, com itens ajustados às suas demandas específicas^(3,63)^; o Qualidade de Vida em Voz (QVV), que investiga como a disfonia interfere na qualidade de vida^(3,64)^; o Índice de Fadiga Vocal (IFV), voltado para a identificação da fadiga vocal, especialmente em contextos de uso prolongado da voz^(24,62,65)^; e o *Evaluation of the Ability to Sing Easily* (EASE-BR), que avalia o conforto e a facilidade na emissão vocal durante o canto^(24,62,66)^. Embora sejam instrumentos amplamente utilizados na prática clínica, esses PROMs não foram desenvolvidos especificamente para avaliar a autopercepção do condicionamento vocal em cantores, uma vez que seu foco principal não está nesse aspecto.

Para garantir que uma medida reflita com precisão o domínio de saúde ao qual se destina, é essencial que seu construto esteja bem definido, assegurando validade e confiabilidade dos dados obtidos^(67,68)^. Essa característica é fundamental na avaliação da eficácia dos programas de condicionamento vocal. PROMs com construtos bem estabelecidos podem ser integrados à prática clínica, auxiliando na tomada de decisões, no acompanhamento do treinamento vocal e no monitoramento da evolução do condicionamento vocal. Além disso, permitem a obtenção de dados em tempo real, favorecendo a personalização das intervenções e a otimização dos resultados^(69)^. A validade de construto de um PROM é um elemento essencial para sua aplicabilidade em diferentes contextos clínicos^(69-71)^. Instrumentos com construtos bem definidos possibilitam um atendimento mais centrado no paciente, pois capturam informações relevantes e alinhadas às suas necessidades, contribuindo para um cuidado mais eficaz e satisfatório^(67-71)^.

Os autores dos estudos participantes desta revisão apontaram limitações e/ou implicações para o futuro a respeito de suas pesquisas. Dentre as implicações podemos destacar: tópicos que estudos futuros poderiam abordar, tais quais: os benefícios de um regime de exercícios mais personalizado e seus efeitos nos parâmetros vocais^(30)^, os requisitos de dose e força vocal para desempenho com a ajuda da amplificação^(26)^, efeitos de um treinamento a longo prazo e a eficácia do treinamento muscular respiratório nos parâmetros acústicos^(29)^.

Com relação as limitações encontradas nos estudos podemos citar: equipamentos utilizados que limitaram a verdadeira mensuração de medidas pretendidas, o nível de experiência dos cantores que dificultou verificar se as pequenas alterações observadas foram, de fato, resultado do programa de treinamento proposto ^(27)^, dificuldades inerentes ao momento da pandemia quando os cantores não estavam atuando da mesma forma como antes^(24)^. Ausência de imagem laríngea, tamanho de amostra reduzido e ausência de avaliações de acompanhamento também foram apontadas como limitações^(23)^.

O presente estudo levantou dados importantes que evidenciam uma carência de maior acurácia e especificidade quanto às características das pesquisas, especialmente sobre os aspectos relacionados à execução dos exercícios. Podem ser citadas algumas limitações, como a ausência de informações importantes sobre variáveis relacionadas com as amostras, os parâmetros de prescrição dos exercícios ou as medidas de resultados, tal qual a heterogeneidade metodológica e das variáveis. Tais imprecisões dificultam a síntese, a análise e a generalização das evidências, expõem uma fragilidade no campo das pesquisas em voz e reforçam a necessidade de pesquisas mais consistentes na área do treino vocal, de modo a apoiar uma prática clínica baseada em evidências para esta população específica, que é tão vulnerável a distúrbios vocais.

## CONCLUSÃO

Há variabilidade nas estratégias de intervenção utilizadas para o treinamento vocal com foco no condicionamento de cantores sem queixas vocais. Abordagens com enfoque em um trabalho simultâneo entre respiração e voz se destacaram, em que exercícios de força muscular respiratória e os de trato vocal semiocluído (ETVSO), especialmente com dispositivos de tubos e canudos, foram os mais utilizados. Além disso, houve maior frequência de estudos que orientaram a execução dos exercícios por número de séries e/ou repetições, embora, na maioria destes, o tempo também tenha sido monitorado. Na maioria, a duração total da intervenção atingiu o tempo mínimo necessário para que as adaptações pretendidas pudessem começar a ocorrer, o que pode levar, em média, de quatro a cinco semanas. A autoavaliação foi a medida de desfecho predominante para analisar os resultados, que se mostraram positivos em todos os estudos. Ademais, o trabalho interdisciplinar entre profissionais de diferentes áreas de atuação se destacou.

## Apêndice A Adaptação da estratégia de busca final para cada base de dados ou fonte de informação

**Table:**

BASE DE DADOS	ESTRATÉGIA DE BUSCA
**PUBMED**	((((((((((((((((((((((((((((((((((((Voice[MeSH Terms]) AND (Singing[MeSH Terms])) OR ("Singing Voice")) OR (Singers)) OR (Singer)) OR ("Healthy Volunteers"[MeSH Terms])) OR ("Healthy Voice") ) AND ("Voice Training"[MeSH Terms]) ) OR ("Vocal Training") ) OR (Exercise[MeSH Terms])) AND (Voice[MeSH Terms]) ) OR ("Vocal Exercises")) OR ("Exercise Physiology")) AND (Voice[MeSH Terms]) ) OR ("Exercise Science")) AND (Voice[MeSH Terms]) )) OR ("Vocal Exercise Physiology") ) OR (Efficiency[MeSH Terms])) AND (Voice[MeSH Terms])) OR ("Muscle Function")) AND (Voice[MeSH Terms])) OR ("Vocal Function")) OR ("Vocal performance")) OR ("Vocal Practice and Performance")) OR ("Vocal efficiency")) OR ("Vocal Economy"))) OR ("Resistance Training"[MeSH Terms]) ) AND (Voice[MeSH Terms]) ) OR ("Strength Training"[MeSH Terms]) ) AND (Voice[MeSH Terms])) OR ("Muscle Fatigue"[MeSH Terms])) AND (Voice[MeSH Terms])
**LILACS/BVS (filtro lilacs)**	((Voice) AND (Singing) OR ("Singing Voice") OR (Singers) OR (Singer) OR ("Healthy Volunteers") OR ("Healthy Voice")) AND (("Voice Training") OR ("Vocal Training") OR (Exercise)) AND ((Voice) OR ("Vocal Exercises") OR ("Exercise Physiology")) AND ((Voice) OR ("Exercise Science")) AND ((Voice) OR ("Vocal Exercise Physiology") OR (Efficiency)) AND ((Voice) OR ("Muscle Function")) AND ((Voice) OR ("Vocal Function") OR ("Vocal performance") OR ("Vocal Practice and Performance") OR ("Vocal efficiency") OR ("Vocal Economy") OR ("Resistance Training")) AND ((Voice) OR ("Strength Training")) AND ((Voice) OR ("Muscle Fatigue")) AND (Voice)
**SCOPUS**	((Voice AND Singing) OR ("Singing Voice" OR Singers OR Singer OR "Healthy Volunteers" OR "Healthy Voice")) AND (("Voice Training" OR "Vocal Training") OR (Exercise AND Voice) OR ("Vocal Exercises") OR ("Exercise Physiology" AND Voice) OR ("Exercise Science" AND Voice) OR ("Vocal Exercise Physiology") OR (Efficiency AND Voice) OR ("Muscle Function" AND Voice) OR ("Vocal Function" OR "Vocal performance" OR "Vocal Practice and Performance" OR "Vocal efficiency" OR "Vocal Economy") OR ("Resistance Training" AND Voice) OR ("Strength Training" AND Voice) OR ("Muscle Fatigue" AND Voice))
**EMBASE**	((((((((('voice'/exp OR 'voice') AND ('singing'/exp OR 'singing') OR 'singing voice' OR singers OR 'singer'/exp OR 'singer' OR 'normal human'/exp OR 'normal human' OR 'healthy voice') AND ('voice training'/exp OR 'voice training') OR 'vocal training' OR 'exercise'/exp OR 'exercise') AND ('voice'/exp OR 'voice') OR 'vocal exercises' OR 'exercise physiology'/exp OR 'exercise physiology') AND ('voice'/exp OR 'voice') OR 'exercise science') AND ('voice'/exp OR 'voice') OR 'vocal exercise physiology' OR 'productivity'/exp OR 'productivity') AND ('voice'/exp OR 'voice') OR 'muscle function'/exp OR 'muscle function') AND ('voice'/exp OR 'voice') OR 'vocal function exercise'/exp OR 'vocal function exercise' OR 'vocal performance' OR 'vocal practice and performance' OR 'vocal efficiency' OR 'vocal economy' OR 'resistance training'/exp OR 'resistance training') AND ('voice'/exp OR 'voice') OR 'muscle fatigue'/exp OR 'muscle fatigue') AND ('voice'/exp OR 'voice')
**WEB OF SCIENCE**	ALL=(((Voice) AND (Singing) OR ("Singing Voice") OR (Singers) OR (Singer) OR ("Healthy Volunteers") OR ("Healthy Voice")) AND (("Voice Training") OR ("Vocal Training") OR (Exercise)) AND ((Voice) OR ("Vocal Exercises") OR ("Exercise Physiology")) AND ((Voice) OR ("Exercise Science")) AND ((Voice) OR ("Vocal Exercise Physiology") OR (Efficiency)) AND ((Voice) OR ("Muscle Function")) AND ((Voice) OR ("Vocal Function") OR ("Vocal performance") OR ("Vocal Practice and Performance") OR ("Vocal efficiency") OR ("Vocal Economy") OR ("Resistance Training")) AND ((Voice) OR ("Strength Training")) AND ((Voice) OR ("Muscle Fatigue")) AND (Voice))
**COCHRANE**	(Voice AND Singing OR Singing Voice OR Singers OR Singer OR Healthy Volunteers OR Healthy Voice) AND (Voice Training OR Vocal Training OR Exercise AND Voice OR Vocal Exercises OR Exercise Physiology AND Voice OR Exercise Science AND Voice OR Vocal Exercise Physiology OR Efficiency AND Voice OR Muscle Function AND Voice OR Vocal Function OR Vocal performance OR Vocal Practice and Performance OR Vocal efficiency OR Vocal Economy OR Resistance Training AND Voice OR Strength Training AND Voice OR Muscle Fatigue AND Voice)
***Google Scholar* (100 primeiros)**	Voice AND Singing AND Voice Training OR Exercise Physiology OR Resistance Training OR Strength Training OR Muscle Fatigue
**ProQuest**	Voice AND Singing AND (“Vocal Training” OR "Voice Training")
**MedRxiv**	Voice AND Singing AND “Voice Training”
**Journal of Singing**	Voice AND Singing AND “Voice Training”
**BDTD**	"Singing Voice" AND "Voice Training" OR “Vocal Training”

## Apêndice B Instrumento para extração dos dados

**Table:**

**Variável**	**Descrição**
**Ano**	Ano em que o artigo foi publicado
**Autoria**	Nomes dos autores que conduziram os estudos
**País de publicação**	País em que o estudo foi publicado
**Título**	Nome designado ao trabalho
**Periódico**	Revista em que o trabalho foi publicado
**Objetivo**	Propósito do estudo
**Delineamento de estudo**	Tipo de estudo classificado de acordo com Medronho^(29)^: experimentais e quase-experimentais, incluindo ensaios clínicos randomizados, ensaios controlados não randomizados, estudos antes e após e estudos e séries de casos
**Características da amostra**	Informações sobre a quantidade de participantes, sexo e idade
**Exercício/técnica utilizada**	Descrição das intervenções que foram utilizadas para alcançar o propósito
**Dosagem**	Dose recomendada em cada estudo
**Frequência de realização dos exercícios**	Periodização do treinamento
**Frequência de realização das sessões**	Periodização das sessões de acompanhamento
**Duração total da intervenção**	Período total em que o treinamento durou
**Profissional que aplicou**	Área de atuação de quem aplicou a intervenção ou conduziu o estudo
**Medidas de desfecho**	Provas e testes utilizados para avaliar os resultados
**Resultados**	Efeitos obtidos após o treinamento proposto
**Conclusão**	Constatação final que está relacionada ao objetivo do estudo
**Limitações/implicações**	Limitações e recomendações trazidas pelos autores do estudo
